# Syd/JIP3 and JNK Signaling Are Required for Myonuclear Positioning and Muscle Function

**DOI:** 10.1371/journal.pgen.1004880

**Published:** 2014-12-18

**Authors:** Victoria K. Schulman, Eric S. Folker, Jonathan N. Rosen, Mary K. Baylies

**Affiliations:** 1 Cell and Developmental Biology, Weill Cornell Graduate School of Medical Sciences, Cornell University, New York, New York, United States of America; 2 Program in Developmental Biology, Sloan-Kettering Institute, New York, New York, United States of America; New York University, United States of America

## Abstract

Highlighting the importance of proper intracellular organization, many muscle diseases are characterized by mispositioned myonuclei. Proper positioning of myonuclei is dependent upon the microtubule motor proteins, Kinesin-1 and cytoplasmic Dynein, and there are at least two distinct mechanisms by which Kinesin and Dynein move myonuclei. The motors exert forces both directly on the nuclear surface and from the cell cortex via microtubules. How these activities are spatially segregated yet coordinated to position myonuclei is unknown. Using *Drosophila melanogaster*, we identified that Sunday Driver (Syd), a homolog of mammalian JNK-interacting protein 3 (JIP3), specifically regulates Kinesin- and Dynein-dependent cortical pulling of myonuclei without affecting motor activity near the nucleus. Specifically, Syd mediates Kinesin-dependent localization of Dynein to the muscle ends, where cortically anchored Dynein then pulls microtubules and the attached myonuclei into place. Proper localization of Dynein also requires activation of the JNK signaling cascade. Furthermore, Syd functions downstream of JNK signaling because without Syd, JNK signaling is insufficient to promote Kinesin-dependent localization of Dynein to the muscle ends. The significance of Syd-dependent myonuclear positioning is illustrated by muscle-specific depletion of Syd, which impairs muscle function. Moreover, both myonuclear spacing and locomotive defects in *syd* mutants can be rescued by expression of mammalian JIP3 in *Drosophila* muscle tissue, indicating an evolutionarily conserved role for JIP3 in myonuclear movement and highlighting the utility of *Drosophila* as a model for studying mammalian development. Collectively, we implicate Syd/JIP3 as a novel regulator of myogenesis that is required for proper intracellular organization and tissue function.

## Author Summary

A common pathology found in numerous cases of muscle diseases, including congenital myopathies and muscular dystrophies, is aberrantly located nuclei within individual multinucleated muscle cells. However, whether or not mispositioned myonuclei are a cause or consequence of muscle disease states is currently debated. Here, we take advantage of the model organism, *Drosophila melanogaster*, which shares the conserved myofiber found in mammalian systems, to identify Syd as a novel regulator of myonuclear positioning. We show that Syd is responsible for mediating the activities of Kinesin and Dynein, two motor proteins that exert forces to pull myonuclei into place. Moreover, we demonstrate that Syd-dependent myonuclear positioning also requires intracellular signaling from the JNK MAPK cascade to direct when and how myonuclei are moved into proper position. This work thus identifies developmental cues that direct proper muscle morphogenesis, suggesting that cases of muscle disease may result from a failure to achieve initial spacing of myonuclei. Supporting this notion, we find that loss of Syd impairs muscle function, but resupplying Syd restores proper myonuclear spacing and muscle function. These findings are particularly important as mispositioned myonuclei gain traction as a potential contributing factor in cases of muscle disease.

## Introduction

The intracellular location of the multiple nuclei within muscle cells has recently gained traction as a potential contributing factor to muscle disease. Improper myonuclear position strongly correlates with muscle disease [1], [2] and muscle weakness [3]–[5]; yet, the mechanisms of myonuclear movement and positioning have only recently begun to emerge.

Recent work in *Drosophila melanogaster* has identified myonuclear positioning as a microtubule-dependent process [4], [6], requiring both Kinesin-1 (Kinesin) and cytoplasmic Dynein (Dynein), the plus- and minus-end directed microtubule motor proteins, respectively [4], [5], [7]. Specifically, two spatially distinct Kinesin- and Dynein-dependent processes position myonuclei [5], [7]. In the first, Kinesin and Dynein exert forces directly on the nucleus: Kinesin extends the front of the myonucleus in the direction of travel, and Dynein is necessary for the retraction of the trailing edge of the myonucleus to complete a translocation step [7]. In the other, Kinesin transports Dynein to the cell cortex near the ends of the muscles where Dynein then pulls microtubule minus-ends and the attached myonuclei into place [5], [7]. Disruption of either pathway leads to mispositioned myonuclei [5], [7], but how Kinesin and Dynein are both used in two spatially segregated mechanisms, and whether their actions in these two different locations are connected or interdependent, is not known.

In many cellular contexts, adaptor proteins specify a variety of motor protein functions. Adaptors either recruit or restrict motors to particular cellular locations [8]–[11], mediate specific motor-cargo interactions [12]-[20], ensure proper temporal and spatial activation of motor function [11], [21]–[23], or enhance motor processivity [24]–[26]. Additionally, adaptor proteins that influence motor function respond to distinct stimuli. Some adaptor proteins regulate motors in response to physical changes of the cell brought on by mechanical strain [27], while others respond to environmental cues via the induction of signaling cascades [13], [28]–[30]. Thus, adaptors are ideal candidates to direct the activities of Kinesin and Dynein during myonuclear positioning.

The JNK signaling cascade is one of three classical mitogen-activated protein kinase (MAPK) cascades conserved across eukaryotes [31]–[37]. Each cascade consists of a MAPK kinase kinase → MAPK kinase → MAPK signaling module that impacts various cellular functions in response to specific stimuli. One function of the JNK MAPK signaling cascade is to phosphorylate JNK-interacting proteins (JIPs), a class of adaptor proteins that regulate Kinesin and Dynein activity in neurons [13], [38]–[41]. While all JIPs interact with both Kinesin and Dynein [14], [21], [38], [39], each adaptor has unique motor-binding domains [13], [38], [42], [43]. JIP1/2 proteins contain a shared motor-binding domain, while JIP3/4 family members have separate Kinesin- and Dynein-binding domains that facilitate binding to both motors simultaneously; thus, different JIPs coordinate motor functions via distinct mechanisms [13], [21], [38], [41]–[44]. Given these features, the JIP proteins could regulate the two Kinesin- and Dynein-dependent pathways governing myonuclear positioning.

To address this possibility, we examined the role of the JIP3 ortholog, Sunday Driver (Syd), during *Drosophila* muscle morphogenesis. We find that Syd responds to the activation of the JNK signaling cascade to regulate myonuclear positioning by specifically promoting Kinesin-dependent localization of Dynein to the muscle ends. Furthermore, we propose that Kinesin and Dynein are both initially perinuclear and that Syd specifies a subset of Kinesin to relocate Dynein to the muscle cell cortex to initiate cortical pulling of myonuclei. Finally, we demonstrate that disrupted JNK signaling or loss of Syd decreases muscle function, indicating that proper regulation of the JNK signaling cascade and the JIP adaptor proteins is not only necessary for intracellular organization but also critical for muscle function.

## Results

### Sunday Driver is expressed in muscle tissue

**Figure 1:**
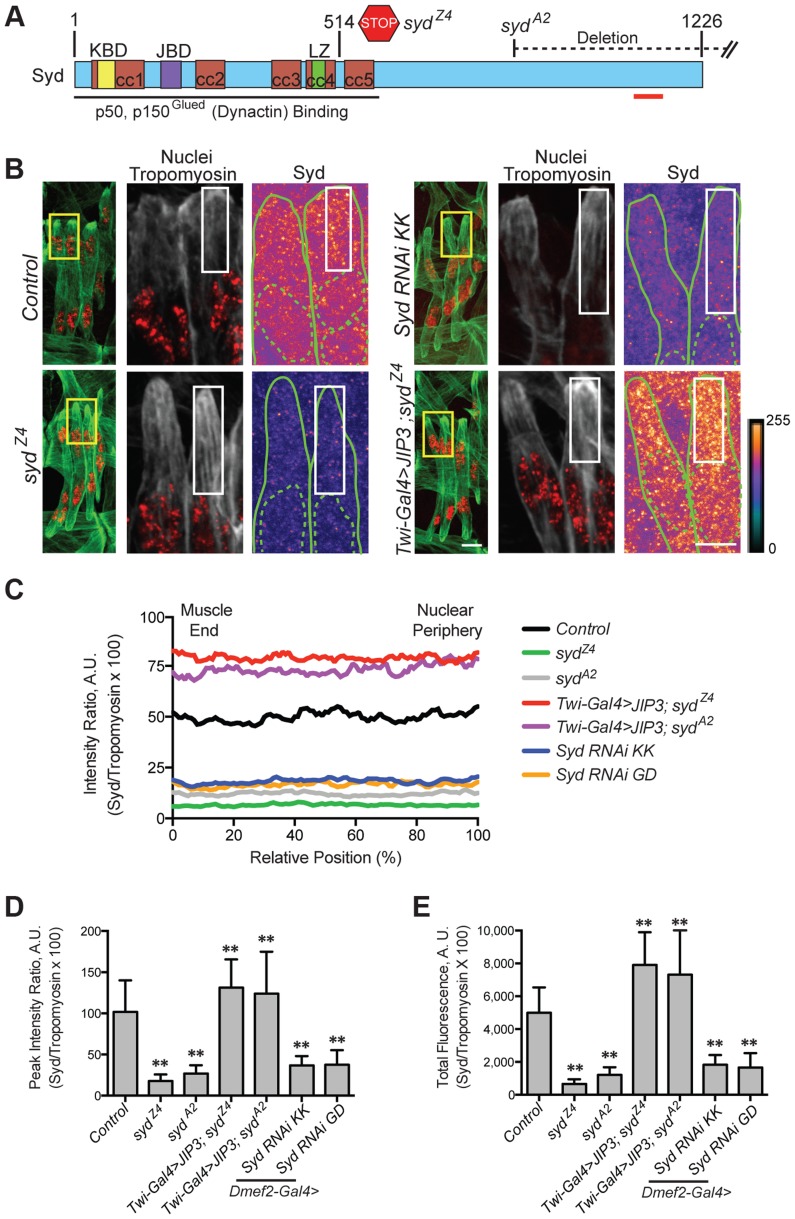
Syd is expressed in muscle tissue. **A**) Diagram of Syd protein based on previous work and physical interaction data [39], [40], [45]. Amino acid number indicated above at distinct points. Yellow, Khc Binding Domain (KBD); purple, JNK Binding Domain (JBD); green, Leucine Zipper (LZ), mediates Klc binding; red, coiled coil (cc) domains 1–5. N-terminus mediates Dynactin binding (LZ for mammalian JIP3 [21]). *syd* alleles are noted [45]. Red line indicates the conserved region of Syd/JIP3 recognized by the C-terminal Syd antibody (S1A Fig.). **B**) (*Left)* Immunofluorescence projection images of the LT muscles in one hemisegment of stage 16 *Drosophila* embryos in the indicated genotypes. Green, Tropomyosin/muscles; red, dsRed/nuclei. Yellow boxes denote regions of higher magnification to the right. Scale bar, 10 µm. (*Middle, Right*) Higher magnification views used for analysis. Grayscale, Tropomyosin; red, nuclei (middle). Syd shown as a heatmap (right) to highlight regions of accumulation. Scale of relative intensities shown at lower right. Solid green lines outline the muscles as noted by Tropomyosin staining. Dotted green lines highlight the nuclei as noted by dsRed staining. White boxes denote regions used for analyses in C-E. Scale bar, 5 µm. **C**) Intensity profile of Syd immunofluorescence relative to Tropomyosin immunofluorescence plotted as a function of position normalized as a percentage of the total distance from the muscle end (left, 0%) to the nearest nucleus (right, 100%). **D**) Average peak intensity values for Syd immunofluorescence. **E**) Average total Syd immunofluorescence, determined by calculating the area under the curves in C. For each genotype in B-E, two LT muscles were measured in each of three hemisegments from ten embryos from at least three independent experiments. All error bars represent standard deviation. **, p<0.01 compared to controls (Student's t-test and ANOVA assessment). A.U., arbitrary units.

We hypothesized that the adaptor protein, Sunday Driver (Syd), may regulate one, or both, of the spatially distinct Kinesin- and Dynein-dependent pathways that impact myonuclear positioning from 1) the nucleus, and 2) the muscle end [5], [7]. In neurons, Syd physically interacts with both Kinesin and Dynein to coordinate motor activity and promote axonal transport (Fig. 1A) [39], [40], [45]. Because Syd has only been studied in the central nervous system (CNS), we immunostained for Syd and quantified the immunofluorescence intensity of the signal specifically in the Lateral Transverse (LT) muscles (Fig. 1B). Importantly, we compared Syd intensity to that of Tropomyosin, which did not significantly vary between genotypes (S1A-B Figs.) and therefore served as an internal immunostaining control. Intensities were plotted as a function of position (Fig. 1C), and both the peak intensity value (Fig. 1D) and the area under the curve (Fig. 1E), indicating the maximum and total fluorescence, respectively, were used as measures of Syd protein levels.

These analyses demonstrated that Syd is highly expressed in the cytoplasm of muscle cells with no discernable regions of distinct accumulation (Fig. 1B–E, S1A–B Figs.), similar to observations of Syd in other tissues [39], [45]. This signal was lost in two different *syd* mutants (*syd^A2^* and *syd^Z4^*) and in embryos that were depleted of Syd via GAL4/UAS-mediated [46] expression of *Syd-RNAi* specifically in the muscles (Fig. 1A–E, S1A–B Fig.). These analyses indicate that Syd is expressed in muscle tissue at the correct time and place to influence myonuclear positioning.

### Syd is required for myonuclear positioning

**Figure 2:**
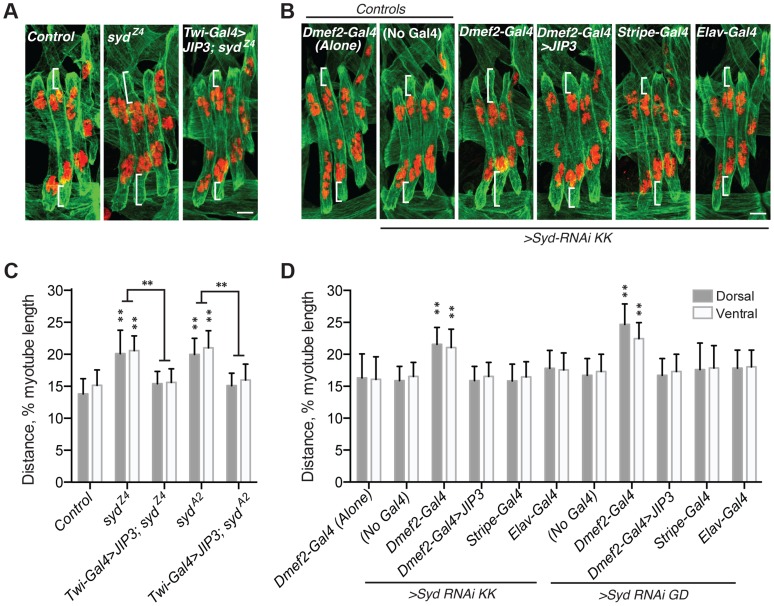
Syd is required to position myonuclei. **A–B**) Immunofluorescence projection images of the LT muscles in one hemisegment of stage 16 *Drosophila* embryos of the indicated genotypes. Green, Tropomyosin/muscles; red, dsRed/nuclei. White brackets indicate the distance from the dorsal and ventral muscle ends and the nearest nucleus used for quantification of myonuclear position in C–D. Scale Bar, 10 µm. **A**) *syd* mutants **B**) *Syd-RNAi* expressed under the control of the indicated GAL4 driver. **C–D**) Histograms indicating the shortest distance between the indicated LT muscle end (Dorsal, grey; Ventral, white) and the nearest nucleus normalized for muscle length for the genotypes indicated in A–B. For each genotype in A–D, all four LT muscles were measured in each of three hemisegments from ten embryos from at least three independent experiments. All error bars represent standard deviation. **, p<0.01 compared to controls, ‘Dmef2-Gal4 Alone,’ and ‘No Gal4,’ unless otherwise noted by brackets (Student's t-test and ANOVA assessment).

To determine whether myonuclear positioning was disrupted in *syd* mutants, we measured the distance between the myonuclei and the ends of the muscles at embryonic stage 16 (16 h After Egg Laying, AEL) as previously described [5], [7]. At this developmental stage, the myonuclei in controls reside in two groups near the dorsal and ventral ends of the muscles. However, in both *syd^Z4^* and *syd^A2^* mutants, the myonuclei were located significantly further from the muscle ends relative to controls (Fig. 2A,C), suggesting that Syd is required for proper myonuclear positioning.

To confirm that the role of Syd in myonuclear positioning is muscle autonomous, we assessed myonuclear position when *Syd-RNAi* was expressed in the muscles (*Dmef2-Gal4)*, tendons (*Stripe-Gal4*), or CNS (*Elav-Gal4*). Expression of *Syd-RNAi* specifically in the muscles phenocopied *syd* mutants, while RNAi-mediated depletion of Syd in either the tendons or the CNS had no effect on myonuclear position (Fig. 2B,D). These data indicate that Syd impacts myonuclear position in a muscle autonomous manner.

Finally, we rescued *syd*-related myonuclear positioning defects with GAL4/UAS-mediated expression of JIP3 (also known as mSyd2 and JSAP1). JIP3 is the mammalian ortholog of Syd, bearing 69% similarity and 42% identity to *Drosophila* Syd. Expressing JIP3 in the mesoderm using *twist-Gal4* (Fig. 1A–E, S1A–B Fig. and S2A Fig.) rescued myonuclear positioning in *syd* mutants (Fig. 2A,C). Additionally, because mammalian JIP3 and *Drosophila* Syd do not align in the regions targeted by *Syd-RNAi* (S2B–E Fig.), we co-expressed JIP3 and *Syd-RNAi* in the muscles and rescued the effects of RNAi-mediated Syd depletion on myonuclear position (Fig. 2B,D). Collectively, these data emphasize the conservation of this protein across species and confirm that Syd is required in muscle tissue for proper positioning of myonuclei.

### Syd regulates Kinesin- and Dynein-dependent myonuclear positioning

**Figure 3:**
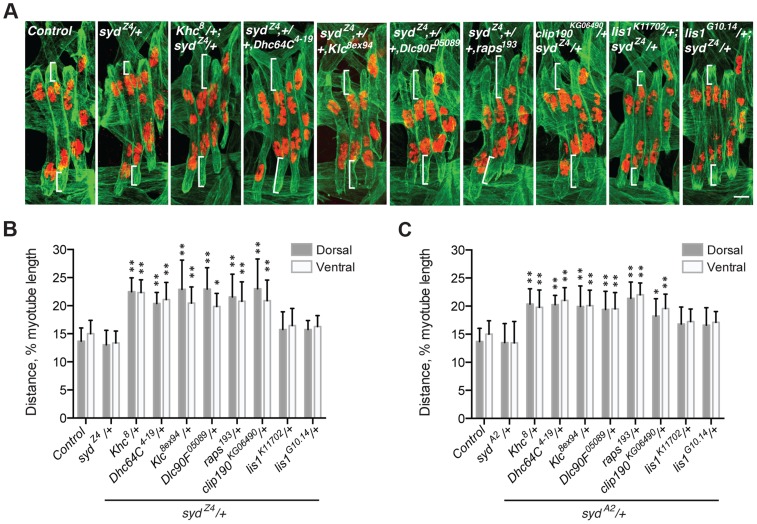
*syd* genetically interacts with factors required for myonuclear positioning. **A**) Immunofluorescence projection images of the LT muscles in one hemisegment of stage 16 *Drosophila* embryos of the indicated genotypes in which the *syd* allele was maternally provided. Green, Tropomyosin/muscles; red, dsRed/nuclei. Scale Bar, 10 µm. **B**) Histogram indicating the shortest distance between the indicated LT muscle end (Dorsal, grey; Ventral, white) and the nearest nucleus normalized for muscle length for the *syd^Z4^* genetic interactions in A. **C**) Identical experiment as in A–B using the *syd^A2^* allele in genetic interactions. For each genotype in A–C, all four LT muscles were measured in each of three hemisegments from ten embryos from at least three independent experiments. All error bars represent standard deviation. *, p<0.05; **, p<0.01 compared to wild-type and heterozygous controls (Student's t-test and ANOVA assessment).

Myonuclear positioning defects in *syd* mutants are similar to those in both *Kinesin heavy chain* (*Khc*) and *Dynein heavy chain* (*Dhc64C*) homozygous mutants [4], [5], [7]. Given that Syd physically interacts with Kinesin and Dynein [39], [40], [45], we tested whether Syd genetically interacts with Kinesin and/or Dynein during myonuclear positioning by examining doubly heterozygous embryos. Importantly, the myonuclei are properly positioned in embryos heterozygous for *Khc^8^* (null), *Dhc64C^4-19^* (null), and either *syd* allele (S3A Fig.) [7]. However, double heterozygotes of *Khc^8^/+; syd/+* and *Dhc64C^4-19^,+/+,syd* exhibited mispositioned myonuclei (Fig. 3A–C), suggesting that Syd regulates myonuclear positioning in a Kinesin- and Dynein-dependent manner. Reciprocal crosses to control for maternal loading effects produced identical results (S3B–C Fig.). These data suggest that Kinesin, Dynein, and Syd work in a common pathway to position myonuclei.

**Figure 4:**
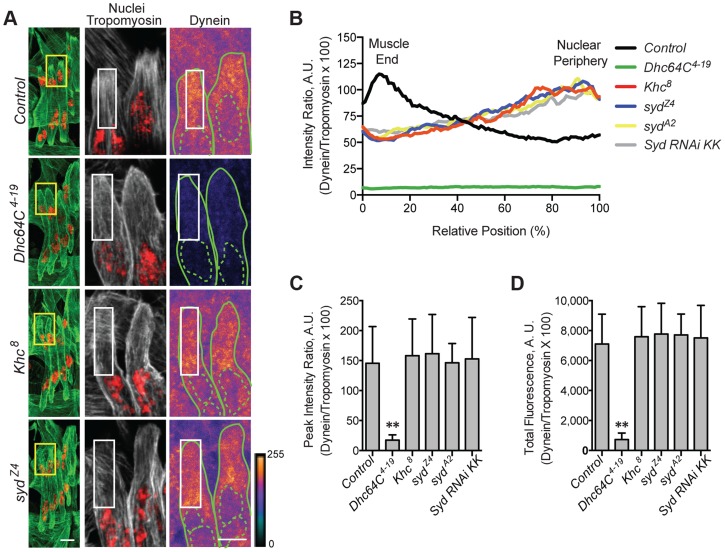
Syd is required for Dynein localization. **A**) (*Left)* Immunofluorescence projection images of the LT muscles in one hemisegment of stage 16 *Drosophila* embryos for the indicated genotypes. Green, Tropomyosin/muscles; red, dsRed/nuclei. Yellow boxes denote regions of higher magnification to the right used for analysis. Scale bar, 10 µm. (*Middle, Right*) Grayscale, Tropomyosin/muscles; red, dsRed/nuclei (middle); Heatmap, Dynein (right). Intensity scale, lower right. Solid and dotted green lines note the perimeter of the muscles and nuclei, respectively. White boxes denote regions used for analyses in B–D. Scale bar, 5 µm. **B**) Intensity profile of Dynein/Tropomyosin immunofluorescence plotted as a function of normalized position (muscle end, left, 0%; nucleus, right, 100%). **C**) Average peak intensity for Dynein immunofluorescence. **D**) Average total Dynein immunofluorescence. For each genotype in A–D, two LT muscles were measured in each of three hemisegments from ten embryos from at least three independent experiments. All error bars represent standard deviation. **, p<0.01 compared to controls (Student's t-test and ANOVA assessment). A.U., arbitrary units.

Adaptors often regulate motors by modulating motor localization. We therefore examined Kinesin (S4A–D Fig.) and Dynein (Fig. 4A–D) localization in control and *syd* mutant embryos, focusing on Kinesin concentrated near the nucleus and Dynein accumulation at the muscle ends, as we previously identified these localization patterns as necessary for proper myonuclear positioning [5], [7]. The intensity of Kinesin or Dynein immunofluorescence was measured relative to that of Tropomyosin, which remained constant between genotypes (S1C–D Fig and S4E–F Fig.), and values were plotted as a function of position. The location of peak intensity indicated where the motor was enriched, while the amplitude of peak intensity and the integrated total fluorescence approximated protein levels. The location and levels of Kinesin immunofluorescence were similar in both controls and *syd* mutants (S4A–F Fig.), suggesting that Kinesin is properly localized near the myonuclei in *syd* mutants. In contrast, Dynein was reduced at the ends of the muscles in *syd* mutants compared to controls (Fig. 4A). This reduction was not due to a decrease in the amount of Dynein present (Fig. 4C,D), but rather, the distribution of Dynein was shifted towards the nucleus in *syd* mutants (Fig. 4B). Consistent with previous reports [7], Dynein was similarly mislocalized in *Khc^8^* mutants (Fig. 4A–D). Together, these data suggest that Syd does not affect Kinesin localization but is required for Kinesin-dependent localization of Dynein to the muscle ends in *Drosophila*.

Importantly, inefficient transport of Dynein is not due to defects in the microtubule network, as gross microtubule organization is comparable to controls in both *Khc^8^* [7] and *syd* mutants (S5A–C Fig.). Therefore, we hypothesized that, as a result of its ability to bind to Kinesin and Dynein [39], [40], [45], Syd mediates an association between the two motors to facilitate Kinesin-dependent localization of Dynein to the cell cortex at the muscle ends to promote proper myonuclear positioning.

**Figure 5:**
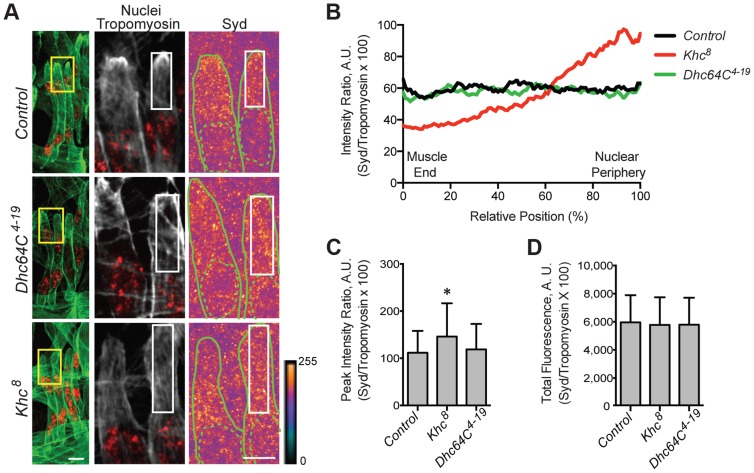
Kinesin is required for Syd localization. **A**) (*Left)* Immunofluorescence projection images of the LT muscles in one hemisegment of stage 16 *Drosophila* embryos for the indicated genotypes. Green, Tropomyosin/muscles; red, dsRed/nuclei. Yellow boxes denote regions of higher magnification to the right used for analysis. Scale bar, 10 µm. (*Middle, Right*) Grayscale, Tropomyosin/muscles; red, dsRed/nuclei (middle); Heatmap, Syd (right). Intensity scale, lower right. Solid and dotted green lines note the perimeter of the muscles and nuclei, respectively. White boxes denote regions used for analyses in B–D. Scale bar, 5 µm. **B**) Intensity profile of Syd/Tropomyosin immunofluorescence plotted as a function of normalized position (muscle end, left, 0%; nucleus, right, 100%). **C**) Average peak intensity of Syd immunofluorescence. **D**) Average total Syd immunofluorescence. For each genotype in A–D, two LT muscles were measured in each of three hemisegments from ten embryos from at least three independent experiments. All error bars represent standard deviation. **, p<0.01 compared to controls (Student's t-test and ANOVA assessment). A.U., arbitrary units.

This hypothesis implies that the intracellular distribution of Syd would be disrupted in *Khc^8^* mutants but unaffected in *Dhc64C^4–19^* mutants. Indeed, Syd was aberrantly enriched near the nucleus in *Khc^8^* mutants, but Syd was found throughout the cytoplasm in controls and *Dhc64C^4–19^* mutants (Fig. 5A–D, S1E–F Fig.). These data argue that the localization of Syd is Kinesin-dependent, but Dynein-independent.

### Syd affects cortical pulling of myonuclei

These data are consistent with a role for Syd in the cortical pulling mechanism of myonuclear positioning in which Kinesin transports Dynein to the muscle ends, and cortically-anchored Dynein pulls microtubules and the attached nuclei towards the muscle ends [5], [7]. To determine whether Syd functions in this pathway, we tested whether Syd functionally interacts with factors shown to be required for this pathway: Dynein light chain (Dlc90F), Raps, and CLIP-190. We also examined Kinesin light chain (Klc), a regulator of the Kinesin motor. Myonuclear position was unaffected in *Klc^8ex94^*, *Dlc90F^05089^*, *raps^193^*, or *clip190^KG06490^* single heterozygous embryos (S3A Fig.). However, embryos doubly heterozygous for *syd* and each of the aforementioned alleles exhibited significantly mispositioned myonuclei (Fig. 3A–C; S3B–C Fig.), suggesting that Syd functionally interacts with each factor in the cortical pathway. Syd did not interact with two different alleles of *lis1*, a regulator of Dynein that does not impact myonuclear positioning in *Drosophila* [5], highlighting the specificity of the observed genetic effects (Fig. 3A–C; S3B–C Fig.). Together, these data suggest that Syd works with Khc, Klc, Dhc64C, Dlc90F, Raps (Pins), and CLIP-190 in a common pathway to position myonuclei. Moreover, these factors impact cortical pulling of myonuclei [5], [7], suggesting that Syd also affects this process.

A role for Syd in one pathway does not exclude its participation in parallel pathways that work towards the same goal. Kinesin and Dynein also exert forces directly on the front and back of myonuclei, respectively, to promote myonuclear movement [7]. This leads to dynamic nuclear shape changes in which myonuclei transition between spherical and elongated nuclear outlines during translocation. Using previously described time-lapse imaging techniques [7], we found that *syd* mutants exhibited myonuclear shape changes similar to controls, and that the myonuclei in *syd* mutants maintained the correct leading edge during translocation (S6A–D Fig.). These data indicate that Syd does not impact the activities of Kinesin and Dynein that influence nuclear dynamics to promote myonuclear positioning [7].

### JNK signaling is required for myonuclear positioning

Together with previously documented physical interaction data [39], [40], [45], these new findings collectively suggest that Syd specifically mediates Kinesin-dependent localization of Dynein to the muscle ends to promote cortical pulling of myonuclei [5], [7]. Syd contains a JNK-binding domain (JBD; Fig. 1A, purple) [40], which mediates binding to JNK/p-JNK [39] and facilitates JNK-dependent phosphorylation of Syd/JIP3 [47]. Furthermore, Syd and related JIPs respond to JNK signaling in neurons to promote cargo binding and influence Kinesin-dependent axonal transport [38], [39], [41], [47], [48]. Thus, we hypothesized that Syd could respond to induction of the JNK signaling cascade to impact myonuclear positioning in muscle tissue.

**Figure 6:**
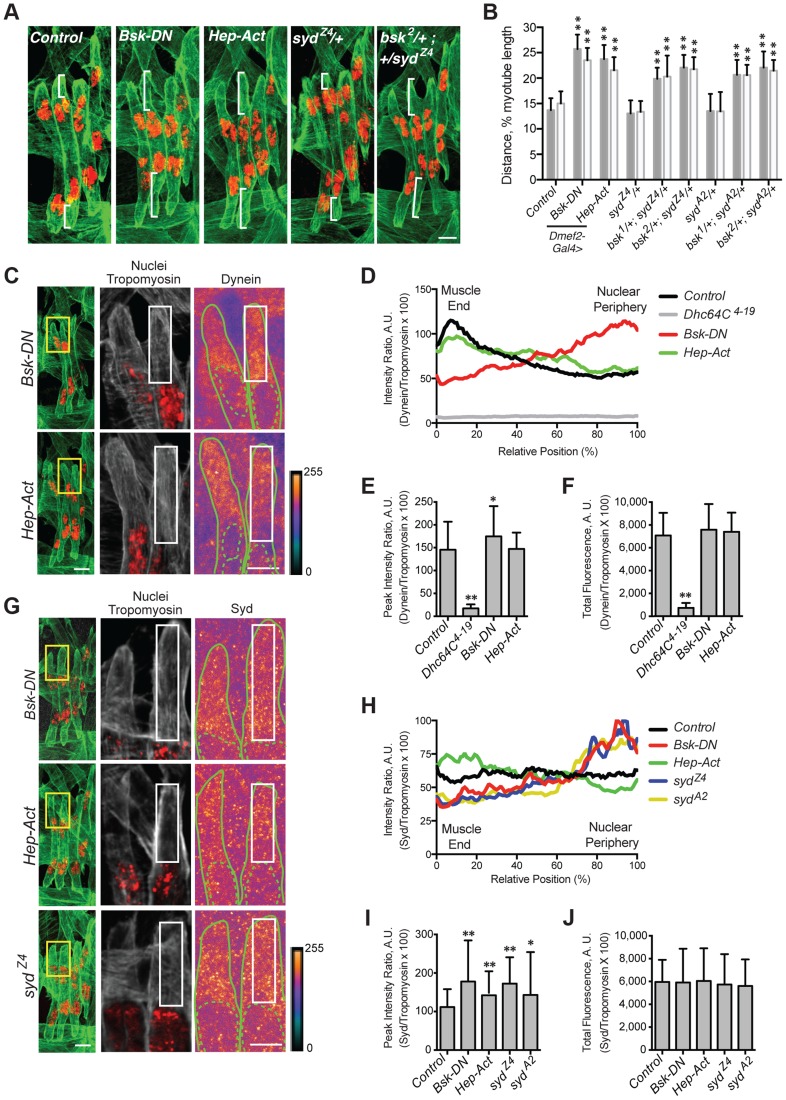
JNK signaling is required for Syd-mediated myonuclear positioning. **A–B**) Identical assay as in Fig. 2. **A**) Stage 16 embryos of the indicated genotypes. Green, Tropomyosin/muscles; red, dsRed/nuclei. Scale Bar, 10 µm. **B**) Histogram indicating the shortest (normalized) distance of white brackets in A. **C–F**) Identical assay as in Fig. 4. **C**) (*Left)* Green, Tropomyosin; red, nuclei. Scale bar, 10 µm. (*Middle, Right*) Grayscale, Tropomyosin; red, nuclei; Heatmap, Dynein. Scale bar, 5 µm. Compare to control in Fig. 4A. **D**) Intensity profile of Dynein/Tropomyosin immunofluorescence plotted as a function of normalized position. **E**) Average peak intensity for Dynein immunofluorescence. **F**) Average total Dynein immunofluorescence. **G–J**) Identical assay as in Fig. 5. **G**) (*Left)* Green, Tropomyosin; red, nuclei. Scale bar, 10 µm. (*Middle, Right*) Grayscale, Tropomyosin; red, nuclei; Heatmap, Syd. Scale bar, 5 µm. Compare to control in Fig. 5A. **H**) Intensity profile of Syd/Tropomyosin immunofluorescence plotted as a function of normalized position. **I**) Average peak intensity for Syd immunofluorescence. **J**) Average total Syd immunofluorescence. For each genotype/experiment, all four LT muscles in A–B and at least two LT muscles in C-J were measured in each of three hemisegments from ten embryos from at least three independent experiments. All error bars represent standard deviation. *, p<0.05; **, p<0.01 compared to controls (Student's t-test and ANOVA assessment). A.U. arbitrary units.

In *Drosophila*, the JNK signaling cascade is composed of Tak1 (MAPKKK), Hep (MAPKK), and Bsk (MAPK), the *Drosophila* ortholog of mammalian JNK [49]. We used the GAL4/UAS system to deplete Tak1, Hep, or Bsk specifically in the muscles, and in each case, the myonuclei were significantly mispositioned further from the muscle ends (S3D Fig.), mimicking *syd*, *Khc*, and *Dhc64C* mutants. Furthermore, muscle-specific expression of dominant negative Bsk (*Bsk-DN*), which cannot be phosphorylated [50], similarly affected myonuclear position (Fig. 6A,B), indicating that loss of JNK signaling disrupts myonuclear positioning.

Interestingly, myonuclear position was also sensitive to the levels of JNK signaling. Overactivation of the cascade with muscle-specific expression of *Hep-Act,* a constitutively active phosphomimetic form of Hep [50], similarly resulted in mispositioned myonuclei (Fig. 6A,B), indicating that disrupted regulation of JNK signaling negatively impacts myonuclear positioning.

We next tested whether JNK signaling requires Syd to position myonuclei. *bsk/+; syd/+* double heterozygotes had mispositioned myonuclei similar to *syd* homozygous mutants and embryos with disrupted JNK signaling (Fig. 6A,B; S3A Fig.), suggesting that Syd and Bsk (JNK) work in a common pathway to position myonuclei. Furthermore, embryos expressing Bsk-DN resembled both *Khc^8^* and *syd* mutants, with both Dynein (Fig. 6C–F, S1G–H Fig.) and Syd (Fig. 6G-J, S1I–J Fig.) aberrantly enriched near the nucleus. These data argue that JNK signaling is required for the cortical pulling pathway of myonuclear positioning.

The effects of overactive JNK signaling were more intriguing. In embryos expressing Hep-Act, Dynein was often properly located at the muscle ends, resembling controls; however, in many instances, Dynein was found more diffusely throughout the cytoplasm (Fig. 6C–F, S1G–H Fig.). When averaged together, these data generated an intermediate distribution curve (Fig. 6D, green). In contrast, Syd consistently becomes aberrantly enriched at the muscle ends in embryos expressing Hep-Act (Fig. 6G–J, S1I–J Fig.). Collectively, these data support that JNK signaling is indeed required to promote Kinesin- and Syd-mediated localization of Dynein to the muscle ends. These data also suggest that improper regulation of JNK signaling affects the maintenance of both Dynein and Syd at the muscle ends.

### The C-terminus of Syd is specifically required for Kinesin/Syd-dependent transport of Dynein to the muscle ends

Syd likely transduces JNK signaling through its JBD to influence Kinesin-dependent transport of Dynein. However, the *syd* alleles result in premature coding truncations that produce N-terminal fragments of Syd (Fig. 1A) [45]. Thus, the JBD as well as the Kinesin- and Dynein-binding domains are present in *syd* mutants; yet, Dynein still fails to localize to the muscles ends, suggesting that the C-terminus of Syd is critical for motor transport. Indeed, immunostaining with an N-terminal Syd antibody demonstrated that truncated Syd was enriched near the nucleus in *syd* mutants despite proper JNK signaling in these genetic backgrounds (Fig. 6G–J, S1I–J Fig.). These data demonstrate that, although no known domains have been identified in the C-terminus of Syd, the C-terminus is required for Kinesin- and JNK signaling-dependent transport of Syd, and therefore Dynein, to the muscle end.

### Syd and JNK signaling are required for muscle function

**Figure 7:**
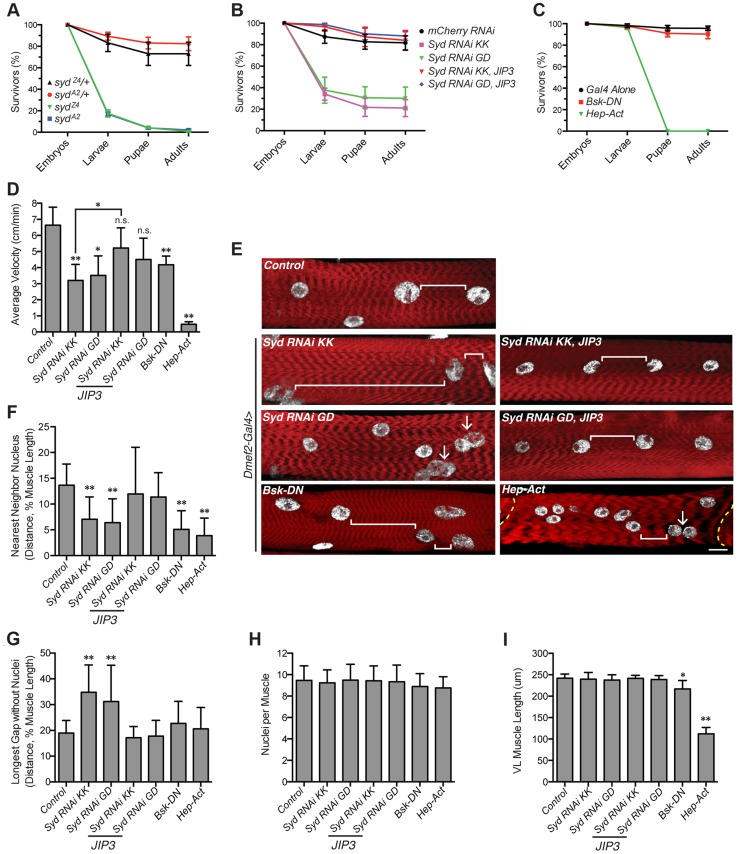
Syd impacts muscle function. **A**) Viability of *syd* mutants. **B**) Viability of embryos expressing *Syd-RNAi* in the muscle tissue driven by *Dmef2-Gal4*. **C**) Viability of embryos with disrupted JNK signaling in muscle tissue via *Dmef2-Gal4* expression. **D**) Average velocity of *Drosophila* larvae as they crawled towards a stimulus. **E**) Immunofluorescence projection images of VL muscles in dissected L3 larvae that were previously used in locomotion assays. Red, Phalloidin/sarcomeres; white, Hoescht/nuclei. White brackets highlight internuclear distance. Arrows denote clumped myonuclei. Yellow dashed line, segment border. Scale bar, 20 µm. **F**) Average distance between each myonucleus and its nearest neighbor normalized for muscle length. **G**) Average length of the longest region of muscle tissue devoid of nuclei normalized for muscle length. **H**) Average number of nuclei per VL muscle. **I**) Average muscle length in the anterior-posterior axis. For each genotype, at least 300 embryos were assessed in A–C and at least 30 larvae were tracked in D from at least three independent experiments each. For each genotype in E–I, three VL muscles (1, 2, and 4) in six hemisegments from five larvae from at least three independent experiments were measured/counted. All error bars represent standard deviation. *, p<0.05; **, p<0.01 compared to controls unless otherwise noted by brackets (Student's t-test and ANOVA assessment). n.s., not significant.

We next investigated whether Syd and/or JNK signaling was necessary for muscle function by quantifying stage L3 larval locomotion using previously described tracking techniques [4], [5], [51]. However, consistent with previous reports [45], only 14–17% of *syd* mutants hatch into larvae (Fig. 7A), and the survivors die soon after hatching, preventing assessments of L3 larval velocity. In contrast, while muscle-specific depletion of Syd using RNAi also leads to decreased viability, 30% of embryos hatch and survive to adulthood, and this lethality can be fully rescued with muscle-specific expression of JIP3 (Fig. 7B). Similarly, while genetic mutations in JNK signaling components leads to embryonic lethality [49], muscle-specific expression of Bsk-DN or Hep-Act does not impair viability at larval stages of development (Fig. 7C). Thus, GAL4/UAS-mediated tissue-specific loss and gain of Syd and JNK signaling was used to assess larval locomotion.

We found that larvae lacking Syd in the muscles crawled significantly slower than controls, but expression of JIP3 in the muscles rescued these locomotion defects (Fig. 7D). Additionally, although overactivation of the cascade was more deleterious, any disruption in JNK signaling negatively affected locomotive ability (Fig. 7D). Importantly, no changes were detected in the muscle innervation sites or the number of boutons at the neuromuscular junctions (S7A–C Fig), both of which, if altered, would be evidence of impaired synaptic transmission [52], [53]. Together, these data indicate that larvae lacking Syd or proper JNK signaling in the muscles exhibit decreased locomotion due to muscle-specific defects independent of communication between the muscles and the CNS.

### Myonuclei are mispositioned in larval muscles

We next examined myonuclear position in the Ventral Longitudinal (VL) muscles by two methods: 1) the Nearest Neighbor analysis described previously [4] to quantify the degree of clustering amongst adjacent myonuclei, and 2) a Longest Gap analysis to measure the greatest longitudinal span of the muscle devoid of nuclei. Importantly, both of these measures were normalized to muscle size to account for variation between genotypes (Fig. 7I). These analyses demonstrated that muscles depleted of Syd exhibit greater degrees of myonuclear clustering and longer spans of muscle tissue devoid of myonuclei compared to controls (Fig. 7E–G). Again, expression of JIP3 in these backgrounds restores all values to control levels (Fig. 7E–G). Importantly, the number of nuclei per VL muscle remains constant across genotypes (Fig. 7H), indicating that clustered nuclei and large muscle regions lacking nuclei are not due to gains or losses of myonuclei.

The effects of disrupted JNK signaling on myonuclear position were more interesting. Both loss and gain of JNK signaling led to significant defects in myonuclear position by Nearest Neighbor assessment (Fig. 7F); however, concomitant defects in muscle length in these backgrounds were also evident (Fig. 7I), indicating that JNK signaling is required for multiple aspects of muscle development. We previously observed a similar developmental relationship between myonuclear positioning and muscle length/growth [5], highlighting that these two processes are closely co-regulated. Interestingly though, we do not observe similar defects in muscle length in larvae lacking Syd, suggesting that Syd functions downstream of JNK signaling to specifically impact myonuclear positioning. Given the well-characterized role of Syd as a JNK interacting protein (JIP) [39]–[41], [54], these data suggest that, while JNK signaling is required for multiple aspects of muscle development, Syd may be a key link that facilitates JNK signaling-mediated regulation of myonuclear position.

## Discussion

**Figure 8:**
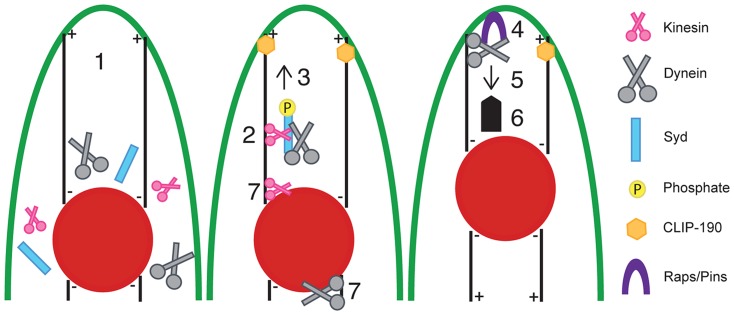
Model of Syd-dependent myonuclear positioning. Syd works in the cortical pulling pathway of myonuclear positioning, which requires cellular transport between the myonuclei (red) and the end of the muscle (green outline). (**1**) Kinesin, Dynein, and Syd are cytoplasmic proteins. Syd mediates (**2**) complex formation between Kinesin and Dynein and (**3**) promotes Kinesin-dependent transport of Dynein to the muscle ends in a JNK signaling-dependent manner. Microtubules contact the cell cortex in a CLIP-190-dependent manner [5]. At the muscle end (**4**) Raps/Pins anchors Dynein to the cell cortex [5]. The Dynein motor becomes active (**5, thin arrow**) and the net result (**6, thick arrow**) of cortically stabilized Dynein activity ultimately pulls the myonuclei into proper position. Syd may also work to initially organize Kinesin and Dynein near the nucleus (**7**) to specify a population of Kinesin to relocate Dynein out to the muscle ends (**2**) while the remaining Kinesin and Dynein exert forces on the nucleus to promote nuclear dynamics (**7**). In this manner, both mechanisms of myonuclear positioning work together to move myonuclei into proper position.

We have used the *Drosophila* musculature to elucidate the cellular mechanisms and signaling pathways that impact myonuclear position *in vivo*. The stereotypic distribution of evenly spaced myonuclei is disrupted in embryos and larvae mutant for Sunday Driver (Syd), an adaptor protein known to regulate Kinesin and Dynein activity in neurons [39], [40], [45]. Here, we show that Syd is expressed and required in muscle tissue to regulate motor activity during myonuclear positioning. Moreover, while Kinesin and Dynein are known to influence myonuclear position via two spatially distinct mechanisms [5], [7], we demonstrate that Syd specifically regulates these motors in the context of cortical pulling at the muscle end without affecting motor activity at the nuclear surface. Syd is a member of the JNK-interacting protein (JIP) family, and we demonstrate that JNK signaling is required for Kinesin- and Syd-dependent localization of Dynein to the muscle ends, which facilitates Dynein-based cortical pulling of myonuclei (Fig. 8). Moreover, in the absence of Syd, both Kinesin and Dynein accumulate near the nucleus, where the motors are known to influence nuclear shape changes/dynamics to promote myonuclear translocation. Syd has no impact on nuclear dynamics; thus, we propose that, via instructive JNK signaling, Syd specifies and activates a population of Kinesin at the nucleus to relocate Dynein to the muscle ends to initiate cortical pulling of myonuclei (Fig. 8, numbers 2 and 7). Finally, we show that loss of Syd and disruption of JNK signaling impairs locomotive ability, indicating that Syd-dependent myonuclear positioning is critical for muscle function.

The finding that JNK signaling is required for this process is novel and significant. Prior to this study, work to elucidate the process of myonuclear positioning focused on identifying factors required for the physical mechanics of moving myonuclei through the cell. However, the present work newly identifies intracellular signals that regulate these activities. Specifically, we show that JNK signaling is required for Kinesin- and Syd-dependent localization of Dynein to the muscle ends. What remains unclear is whether JNK-mediated phosphorylation of Syd [39], [47] induces/permits Dynein binding to Kinesin or if a trimolecular Kinesin-Syd-Dynein complex forms prior to JNK signaling-dependent activation of Kinesin motor activity, which, in turn, transports Dynein to the muscle ends and leads to cortical pulling and proper positioning of myonuclei.

Regardless, our data are consistent with Syd being necessary to relay this instructive JNK signal. Specifically, the C-terminus of Syd is critical for this transport, as Kinesin, Dynein, and truncated Syd are all found near the nucleus in *syd* mutants, which express N-terminal fragments of Syd. This finding was initially unexpected because the minimal domains of Syd required for binding to Kinesin, Dynein, and JNK are all located in the N-terminus (Fig. 1A) [40]. Furthermore, the N-terminus of Syd is sufficient to support Kinesin-based transport *in vitro* [40], [41]. However, the C-terminus of Syd is more highly conserved than the well-annotated N-terminus [45], and biochemical analysis of JIP3 demonstrates that upstream components of the JNK MAPK signaling pathway bind to C-terminal regions of JIP3 [47], [54]. Consistent with these data, we show that mutants lacking the C-terminus of Syd fail to promote proper transport of Dynein to the muscle ends despite normal JNK signaling in these backgrounds. This argues that both the N- and C-termini of Syd are necessary to relay instructive cues from the JNK signaling cascade, which leads to proper myonuclear positioning.

Interestingly, while JNK signaling is indeed necessary for myonuclear positioning, we also demonstrate that constitutively active JNK signaling impairs this process. Despite the inconsistent phenotype, overall decreased amounts of Dynein are found at the muscle ends when Hep-Act is expressed in the muscles. This suggests that overactive JNK signaling either mildly impairs Dynein transport, or alternatively, prevents the maintenance of Dynein at the muscle end. We favor the latter interpretation based on the observation that increased levels of Syd are found at the muscle end when JNK signaling is overactivated. This suggests that efficient transport, and likely excess transport, indeed occurs in embryos expressing Hep-Act. This activity would transport Dynein to the muscle ends; thus, overactive JNK signaling likely inhibits the maintenance of Dynein at this location.

It is not clear how this inhibition could occur; however, one possibility is that failure to dephosphorylate Syd may impair the ability of Dynein to associate with ARF6-GTP, a membrane-bound protein that binds to JIP3/4 proteins [21]. ARF6-GTP and Klc compete for binding to JIP3, and phosphorylated JIPs preferentially bind and activate Kinesin [38]. However, JIP3–ARF6-GTP interactions enhance binding between ARF6-GTP and Dynactin, a well-known Dynein-interacting protein [21]. Thus, in our model, once the Kinesin-Syd-Dynein complex reaches the cell cortex, perhaps Syd must lose the JNK signal and become dephosphorylated to dissociate from Kinesin and bind to ARF6-GTP. This may facilitate hand-off of Dynein to ARF6-GTP, which would aid in securing Dynein to the muscle end.

Although the details of this final step remain unclear, our model (Fig. 8) likely represents a common mechanism by which Syd and other JIP3 orthologs act. Analogous to the proposed manner in which Syd selects certain Kinesin complexes to initiate transport, the *C. elegans* ortholog of Syd/JIP3, UNC-16, was found to exhibit similar gatekeeper characteristics by designating specific complexes to initiate axonal transport in neurons [55]. Furthermore, once specified, UNC-16 also mediates Kinesin-dependent transport of Dynein to the ends of nerve processes [14], [48]. Similarly, mammalian JIP proteins mediate Kinesin-driven transport of many large cargoes, including Dynein [21],[38],[41]. Finally, Syd, UNC-16, and JIP3 directly bind to Kinesin heavy and light chains, and all three orthologs bind to p50 and p150^Glued^, components of the Dynactin complex, a well-known regulator of Dynein [14], [21], [22], [39], [40]. Together with our data, these observations collectively suggest that this mechanism of JIP3-mediated motor coordination (Fig. 8) is well conserved across species and tissues.

These findings are interesting given that Kinesin and Dynein can directly interact *in vitro* [56]; however, adaptors such as Syd and other JIP proteins are likely required to mediate motor protein interactions to direct specific motor functions *in vivo*. Consistent with this notion, structural differences between the JIP1/2 subfamily [13], [42] and the JIP3/4 subfamily of proteins [13], [21], [43], [54] impact how each JIP binds to Kinesin and Dynein and influences motor activity [14], [21], [38], [41]. Although these different JIPs can cooperate towards a single goal [57], they often have unique roles and cannot fully compensate for loss of another *in vivo* [20], [22], [30], [58], [59].

In *Drosophila*, Syd is the only ortholog of the JIP3/4 family of proteins. Similarly, there is only one ortholog of the JIP1/2 family of proteins, Aplip1. Thus, in the context of myonuclear positioning, it is tempting to speculate that while Syd modulates motor activity to promote cortical pulling of myonuclei, perhaps Aplip1 affects the ability of the motors to regulate nuclear dynamics. Furthermore, perhaps JNK signaling is the necessary switch that shifts motor function from one mechanism to the other. Indeed, work in *Drosophila* neurons shows that activation of the JNK signaling cascade disrupts the association between Aplip1 (JIP1) and Kinesin [30], and here we show that JNK signaling is required for coordinated Kinesin-Syd (JIP3) function in the cortical pulling pathway of myonuclear positioning.

Regarding the biological relevance of Syd-dependent mechanisms, we demonstrate that Syd and proper regulation of JNK signaling are critical for muscle function. RNAi-mediated loss of Syd specifically in the muscles leads to decreased larval velocity, and similar locomotive dysfunction is observed in larvae with muscle-specific disruptions in JNK signaling. Consistent with previous reports [4], [5], we show that these locomotive defects are not due to impaired communication with the CNS, but rather correlate with mispositioned myonuclei. These analyses further revealed that muscle-specific disruptions of the JNK signaling cascade additionally impair muscle length/growth, which can also affect locomotion [5]. These findings indicate that JNK signaling is, not surprisingly, necessary for multiple aspects of muscle development. However, that similar concomitant defects are not observed in larvae lacking Syd argues that the role of Syd is more specific to mechanisms of myonuclear positioning. Finally, muscle-specific expression of mammalian JIP3 simultaneously restored myonuclear spacing and rescued larval crawling defects in larvae lacking Syd. These data highlight the high degree of conservation across species, emphasize the muscle autonomous role of Syd, and reiterate the strong correlation between mispositioned myonuclei and decreased muscle output observed previously [4]–[6]. In sum, we have identified that JNK signaling and the motor adaptor, Syd, are required for influencing specific functions of Kinesin and Dynein that lead to proper myonuclear positioning and muscle function, which has significant implications for muscle cell organization, development, and disease.

## Materials and Methods

### *Drosophila* genetics

All stocks were grown under standard conditions. Stocks used: *apterous_ME_-NLS::dsRed* [60], *Df(3L)syd^A2^* and *syd^Z4^* [45], *Khc^8^* [61], *Dhc64C^4−19^* [62], *Klc^8ex94^* [63], *Dlc90F^05089^* [64], *lis1^K11702^* [65], *lis1^G10.14^* [66], *raps^193^* [67], *twist-Gal4* [68], *Dmef2-Gal4* [69], *Stripe-Gal4* (gift from T. Volk), *Elav-Gal4* (gift from E. Lai), *UAS-GFP-JIP3* (this study), *bsk^1^* and *bsk^2^* [49], *UAS-Bsk-DN* and *UAS-Hep-Act* [50]. Bloomington *Drosophila* Stock Center: *clip190^KG06490^* (14493), *UAS-Tak1-RNAi* (33404, 35180), *UAS-Hep-RNAi* (28710, 35210), *UAS-Bsk-RNAi* (31323, 32977, 35594, 36643), *UAS-mCherry-RNAi* (35785). Vienna *Drosophila* RNAi Center: *UAS-Syd-RNAi KK109225* (v101459), *UAS-Syd-RNAi GD12383* (v35346). Mutants were balanced and identified using *CTG* (*CyO, Twi-Gal4, UAS-2xeGFP*) and *TTG* (*TM3, Twi-Gal4, UAS-2xeGFP*) [70].

### Transgenics

Mouse JIP3 construct: pEGFP-C1/mSyd2 (gift from L. Goldstein) [45].

For *Drosophila* rescue experiments, mSyd2 (later re-annotated as JIP3) was amplified from the pEGFP-C1/mSyd2 construct using the following primers and cloned into the pUAST vector containing GFP: mSyd2 Forward: 5′-CACCGAATTCATGGAGATCCAGATGGACGAGGGA-3′; mSyd2 Reverse: 5′-GAATTCCTCAGGGGTGTAGGACACCTGCCA-3′.

All constructs were sequenced and verified, and pUAST-GFP/mSyd2 DNA was injected into *Drosophila* embryos using transposable-element-based insertion methods (Genetic Services, Inc.) to generate flies carrying *UAS-GFP-JIP3* on either chromosome II or III that were used in experiments.

### Immunohistochemistry

Whole mount embryo staining was performed as described [60]. For Dynein localization measurements, embryos were fixed with 10% formalin diluted 1∶1 in heptane for 20 minutes, then rinsed three times in PBS containing 0.3% Triton X-100, then fixed with 4% EM-grade paraformaldehyde in PBS diluted 1∶1 in heptane for 20 minutes. In all cases, embryos were devitellinized by vortexing in a 1∶1::methanol:heptane solution. Larvae were dissected in ice cold HL3.1 as previously described [71] and fixed with 10% Formalin (Sigma, HT501128-4L). Dynein antibody incubations were performed in PBS supplemented with 0.2% BSA and 0.15% Triton X-100. All other antibody incubations were performed in PBS supplemented with 0.1% BSA and 0.3% Triton X-100. Embryos and larvae were mounted in ProLong Gold (Invitrogen) for fluorescent immunostainings. Antibodies were preabsorbed (PA) as described [72] where noted and used at the indicated final dilutions: rabbit anti-dsRed (1∶400, Clontech, 632496), rat anti-Tropomyosin (PA, 1∶500, Abcam, ab50567), mouse anti-GFP (PA, 1∶200, Clontech, 632381), mouse anti-Dhc (1∶50, Developmental Studies Hybridoma Bank), mouse anti-Tubulin (1∶500, Sigma T9026), rabbit anti-Khc (1∶200, Cytoskeleton Inc., AKIN01), mouse anti-Discs large (1∶200, Developmental Studies Hybridoma Bank), rabbit anti-N-terminal-Syd (SN1) and rabbit anti-C-terminal-Syd (7704) (1∶300) (gifts from V. Cavalli and L. Goldstein) [45]. Alexa Fluor 488-, Alexa Fluor 555-, and Alexa Fluor 647-conjugated fluorescent secondary antibodies (1∶200), Alexa Fluor 546-conjugated Phalloidin (1∶100), and Hoechst-33342 (1 µg/1ml) were used for fluorescent stains (Invitrogen). Fluorescence projection images were acquired on a Leica SP5 laser scanning confocal microscope equipped with a 63× 1.4 NA HCX PL Apochromat oil objective and LAS AF 2.2 software unless noted otherwise. Maximum intensity projections of confocal Z-stacks were rendered using Volocity 6.1.1 Visualization software (Improvision). All resulting 2D projection images were cropped using Adobe Photoshop CS6.

### Nuclear position and muscle length measurements

Analysis was performed as described [5].

### Kinesin, Dynein, and Syd localization measurements

High magnification projection images of a set 2 µm depth-size were acquired as described [5], ensuring that the entire Z-stack was acquired from completely within the muscle fiber. Any projection images with a slice including the muscle cell membrane or any area outside the bounds of the muscle were discarded to eliminate variation in background fluorescence. Immunofluorescence intensity was assessed across defined regions using ImageJ (NIH). In all cases, boxed regions were unbiasedly selected in the Tropomyosin/nuclear projection image and transferred to identical regions in other channels. For Kinesin, a box of set dimensions was used. For Dynein and Syd, a box of set width and varying length was used to measure immunofluorescence between the end of the muscle and the nearest nucleus. Immunofluorescence intensity of Kinesin, Syd, or Dynein, was compared to Tropomyosin intensity, and the ratios were multiplied by 100. For Kinesin, average ratios were then plotted as a function of raw position. For Dynein and Syd, the total distance was normalized to 100 to account for myonuclear positioning defects, and average ratios were plotted as a function of normalized position (from muscle end to the nearest nucleus). The area under the curves (total fluorescence) were calculated in Microsoft Excel. The greatest pixel intensity denoted the peak fluorescence intensity.

### Microtubule organization measurements

Analysis was performed as previously described [5] using ImageJ software (NIH) and projection images acquired at a 3X optical zoom on a Zeiss LSM510 laser-scanning confocal microscope equipped with a 63×1.4 NA HCX PL Apochromat oil DIC objective and ZEN 2009 software.

### Time-lapse imaging and nuclear dynamics analysis

Experiments and analyses were performed as described [7].

### Larval behavior, dissections, and analysis

Larval speed was assessed as previously described [5]. Tracked larvae were dissected, stained, and analyzed as described above. Using projection images, internuclear distance was assessed in muscles VL1, VL2, and VL4 using ImageJ (NIH) to measure the distance between each nucleus and the nearest neighboring nucleus. The greatest span of muscle devoid of nuclei was measured in the same manner. All distances were normalized to muscle length (measured from projection images using ImageJ). The number of nuclei per muscle, number of NMJs per muscle, and number of boutons per NMJ were quantified manually using a Leica SP5 laser-scanning confocal microscope with a 20×0.7 NA HCX PL Apochromat oil objective to view the muscles.

### Viability assays

Embryos were collected at 25°C on yeasted apple juice agar plates using overnight lays. Stage 15–16 embryos lacking the balancer (where applicable) were staged by development of the gut and hand-selected for analysis. Embryos were counted, transferred to a lightly yeasted apple juice agar plate, and raised at 22°C overnight. The following day, L1 larvae were counted and transferred to vials of standard fly food at 22°C. Eight to twelve days later, the number of pupal cases and adults present in the vials were quantified. All values were normalized to 100%.

### Alignments

Protein and nucleotide alignments were performed using Clustal Omega and ClustalW2, respectively (EMBL-EBI).

### Statistics

All data sets were analyzed similarly. Error bars represent standard deviations calculated in Excel. Traditional pairwise comparisons using the Student's t-test were used to compare experimental values to control values. Additionally, Analysis of Variance (ANOVA) was used to compare the same data in the context of an entire experiment (three or more values per comparison), taking into account variations in multiple means and standard deviations amongst genotypes within a given experiment. With one-way ANOVA, multiple groups of data were compared simultaneously to determine significance (for example, comparing homozygous mutants to both wild-type and heterozygous controls). Values reaching a threshold of statistical significance in both the Student's t-test and the more stringent ANOVA assessment were considered significant and noted with asterisks in the relevant graphs. All analyses were performed using Prism 6.0.

## Supporting Information

S1 Figure **Tropomyosin levels are similar in tested genotypes.** **A–J**) Validation for the use of Tropomyosin as a reference/internal immunostaining control for comparing changes in immunofluorescence of the protein-of-interest in the experiments detailed in the main text Figs. 1B–E, 4A–D, 5A–D, and 6C–J. **A,C,E,G,I**) Immunofluorescence intensity profile for Tropomyosin plotted as a function of position across the same cellular region examined in the main text Figs. **B,D,F,H,J**) Average total fluorescence of Tropomyosin determined by calculating the area under the curves A, C, E, G, and I, respectively. **A,B**) Controls for differences in Syd immunofluorescence observed in Fig. 1B–E. **C,D**) Controls for differences in Dynein immunofluorescence observed in Fig. 4A–D. **E,F**) Controls for differences in Syd immunofluorescence observed in Fig. 5A–D. **G,H**) Controls for differences in Dynein immunofluorescence observed in Fig. 6C–F. **I,J**) Controls for differences in Syd immunofluorescence observed in Fig. 6G–J. For each genotype in A–J, two LT muscles were measured in each of three hemisegments from ten embryos from at least three independent experiments. All error bars represent standard deviation. Values are not significant by Student's t-test or ANOVA assessment. A.U., arbitrary units.
(PDF)

S2 Figure **Syd protein and DNA alignments.** **A**) Protein alignment of Syd to mammalian JIP3 using Clustal Omega. Amino acid number indicated at right. Red box indicates the conserved region recognized by the C-terminal Syd/JIP3 antibody used in main text Fig. 1 to detect both proteins. *, denotes identical residues; dots, similar residues. **B**–**E**) *Syd* or mammalian *JIP3* DNA sequences aligned to either *Syd-RNAi KK* or *Syd-RNAi GD* using Clustal W2. Nucleotide number indicated at right. *, denotes nucleotide matches. **B**) Alignment of *Syd* to *Syd-RNAi KK*. **C**) Alignment of *Syd* to *Syd-RNAi GD*. **D**) Alignment of *JIP3* to *Syd-RNAi KK*. **E**) Alignment of *JIP3* to *Syd-RNAi GD*.
(PDF)

S3 Figure **Supporting genetic assays.** **A-D**) Analysis of myonuclear position by measuring the shortest distance between the LT muscle ends (Dorsal, grey; Ventral, white) and the nearest nucleus normalized for muscle length using confocal projection imagess of stage 16 embryos immunostained for Tropomyosin/muscles (green) and dsRed/nuclei (red). **A**) Single heterozygous embryos for the listed alleles. **B**) Doubly heterozygous embryos of *syd^Z4^* and factors involved in muscle development in which the *syd^Z4^* allele was paternally provided. **C**) Identical to B using the *syd^A2^* allele. **D**) Multiple *UAS-RNAi* constructs targeting JNK signaling module components expressed in muscles using *Dmef2-Gal4*. For each genotype in A–D, all four LT muscles were measured in each of three hemisegments from ten embryos from at least three independent experiments. All error bars represent standard deviation. *, p<0.05; **, p<0.01 compared to controls (Student's t-test and ANOVA assessment).
(PDF)

S4 Figure **Syd is not required for Kinesin localization.** **A**) (*Left*) Immunofluorescence projection images of the LT muscles in one hemisegment of stage 16 embryos. Green, Tropomyosin/muscles; red, dsRed/nuclei. Yellow boxes identify regions of higher magnification shown to the right. Scale bar, 10 µm. (*Middle, Right*) High magnification views of Kinesin immunofluorescence near the nuclei (grayscale). Kinesin shown as a heatmap to highlight regions of accumulation (right). Scale of relative intensities shown at lower right. White boxes denote regions used for immunofluorescence analysis of Kinesin localization. Scale bar, 5 µm. **B**) Intensity profile of Kinesin immunofluorescence relative to Tropomyosin immunofluorescence plotted as a function of position. Zero/left, corresponds to the top end of white boxed regions in A; Right, corresponds to the lower end of white boxed regions in A. **C**) Average peak intensity of Kinesin immunofluorescence. **D**) Average total fluorescence of Kinesin determined by calculating the area under the curves in B. **E–F**) Validation for the use of Tropomyosin, which is similar in all genotypes, as a reference/internal immunostaining control for comparing changes in Kinesin immunofluorescence in B–D. **E**) Immunofluorescence intensity profile for Tropomyosin plotted as a function of position across the same cellular region examined in B. **F**) Average total fluorescence of Tropomyosin determined by calculating the area under the curves in E. For each genotype A–F, two LT muscles were measured in each of three hemisegments from ten embryos from at least three independent experiments. All error bars represent standard deviation. **, p<0.01 compared to controls (Student's t-test and ANOVA assessment). A.U., arbitrary units.
(PDF)

S5 Figure **Syd does not affect gross microtubule organization.** **A**) (*Left*) Immunofluorescence projection images of the LT muscles in one hemisegment of stage 16 embryos. Green, Tropomyosin/muscles; red, dsRed/nuclei; white, Tubulin/microtubules. Yellow boxes identify regions of higher magnification shown to the right. Scale bar, 10 µm. (*Right three panels*) High magnification views of Tropomyosin and Tubulin immunofluorescence shown in grayscale and merged (colors as in A). Scale bar, 5 µm. **B**) Average Tubulin intensity relative to Tropomyosin intensity in the distal 2 µm of the muscle fiber, demonstrating that microtubules reach the muscle end in all genotypes. **C**) Validation for the use of Tropomyosin, which is similar in all genotypes, as a reference/internal immunostaining control for comparing changes in Tubulin immunofluorescence in B. Values represent total Tropomyosin immunofluorescence detected in the distal 2 µm of the muscle fiber. For each genotype in A–C, two LT muscles were measured in each of three hemisegments from ten embryos from at least three independent experiments. All error bars represent standard deviation. Values are not significant by Student's t-test or ANOVA assessment. A.U., arbitrary units.
(PDF)

S6 Figure **Syd does not impact Kinesin- and Dynein-dependent nuclear shape changes and translocation dynamics.** **A**) Kymographs of individual translocating myonuclei in the indicated genotypes with all nuclei moving in the upward direction. Yellow lines indicate the leading edge of the myonucleus and highlight leading edge dynamics during translocation. Blue lines complete the perimeter of the nuclei at individual time-points and are combined with the yellow lines to produce the cartooned shapes to the right to highlight nuclear shape changes over time. Scale bar, 2 µm. **B**) Histogram indicating the average maximum and minimum aspect ratio of myonuclei as they translocated for between 20 min and 1 h. Maximums and minimums were compared individually. **C**) Histogram indicating the number of shape changes individual myonuclei experience per hour. Values were calculated by following individual myonuclei as they moved for between 20 min and 1 hour. **D**) Histogram indicating the percentage of total nuclei that change direction per hour in indicated genotypes. For each genotype in A-D, 100 individual LT muscle nuclei from at least three hemisegments from five different embryos from at least three independent experiments were measured. All error bars represent standard deviation. Values are not significant by Student's t-test or ANOVA assessment.
(PDF)

S7 Figure **NMJs are unaffected in** *syd* **mutants.** **A**) Immunofluorescence projection images of the VL muscles in dissected L3 *Drosophila* larvae. Red, Phalloidin/sarcomeres; green, Discs large/NMJs; white, Hoescht/nuclei. Scale bar, 20 µm. **B**) Number of boutons per NMJ as determined by Discs large staining. **C**) Number of NMJs/innervations per muscle. For each genotype in A–C, three VL muscles (1, 2, and 4) in six hemisegments from five dissected larvae from at least three independent experiments were measured/counted. All error bars represent standard deviation. Values are not significant by Student's t-test or ANOVA assessment.
(PDF)
