# Supplementary figures and images for: Syd/JIP3 and JNK Signaling Are Required for Myonuclear Positioning and Muscle Function
Source: PLoS Genet. 2014 Dec 18;10(12):e1004880. doi: 10.1371/journal.pgen.1004880 (PMC4270490; doi:10.1371/journal.pgen.1004880)

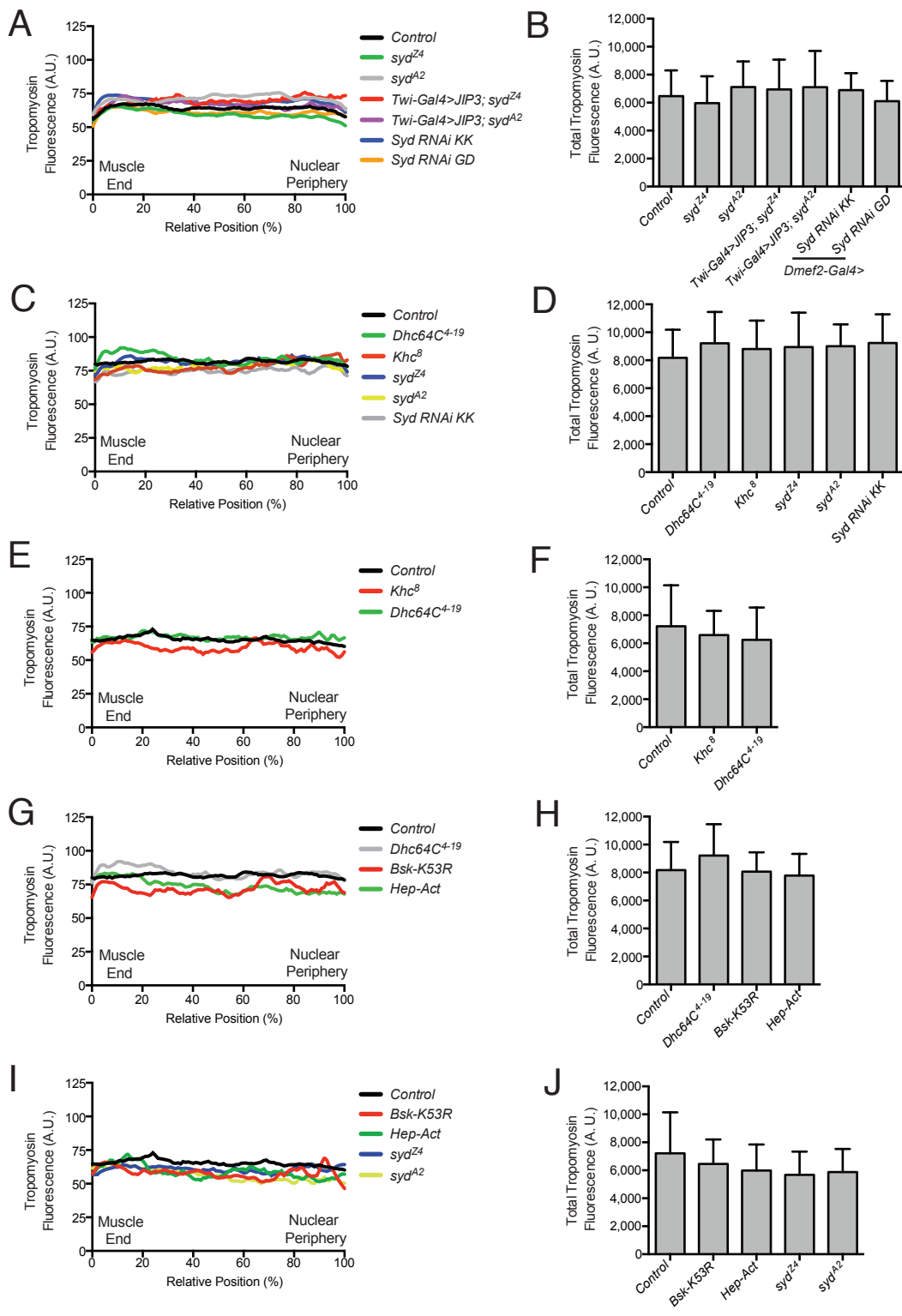

Supplement: S1 Figure — Tropomyosin levels are similar in tested genotypes. A–J) Validation for the use of Tropomyosin as a reference/internal immunostaining control for comparing changes in immunofluorescence of the protein-of-interest in the experiments detailed in the main text Figs. 1B–E, 4A–D, 5A–D, and 6C–J. A,C,E,G,I) Immunofluorescence intensity profile for Tropomyosin plotted as a function of position across the same cellular region examined in the main text Figs. B,D,F,H,J) Average total fluorescence of Tropomyosin determined by calculating the area under the curves A, C, E, G, and I, respectively. A,B) Controls for differences in Syd immunofluorescence observed in Fig. 1B–E. C,D) Controls for differences in Dynein immunofluorescence observed in Fig. 4A–D. E,F) Controls for differences in Syd immunofluorescence observed in Fig. 5A–D. G,H) Controls for differences in Dynein immunofluorescence observed in Fig. 6C–F. I,J) Controls for differences in Syd immunofluorescence observed in Fig. 6G–J. For each genotype in A–J, two LT muscles were measured in each of three hemisegments from ten embryos from at least three independent experiments. All error bars represent standard deviation. Values are not significant by Student's t-test or ANOVA assessment. A.U., arbitrary units. (PDF) [file pgen.1004880.s001.pdf]

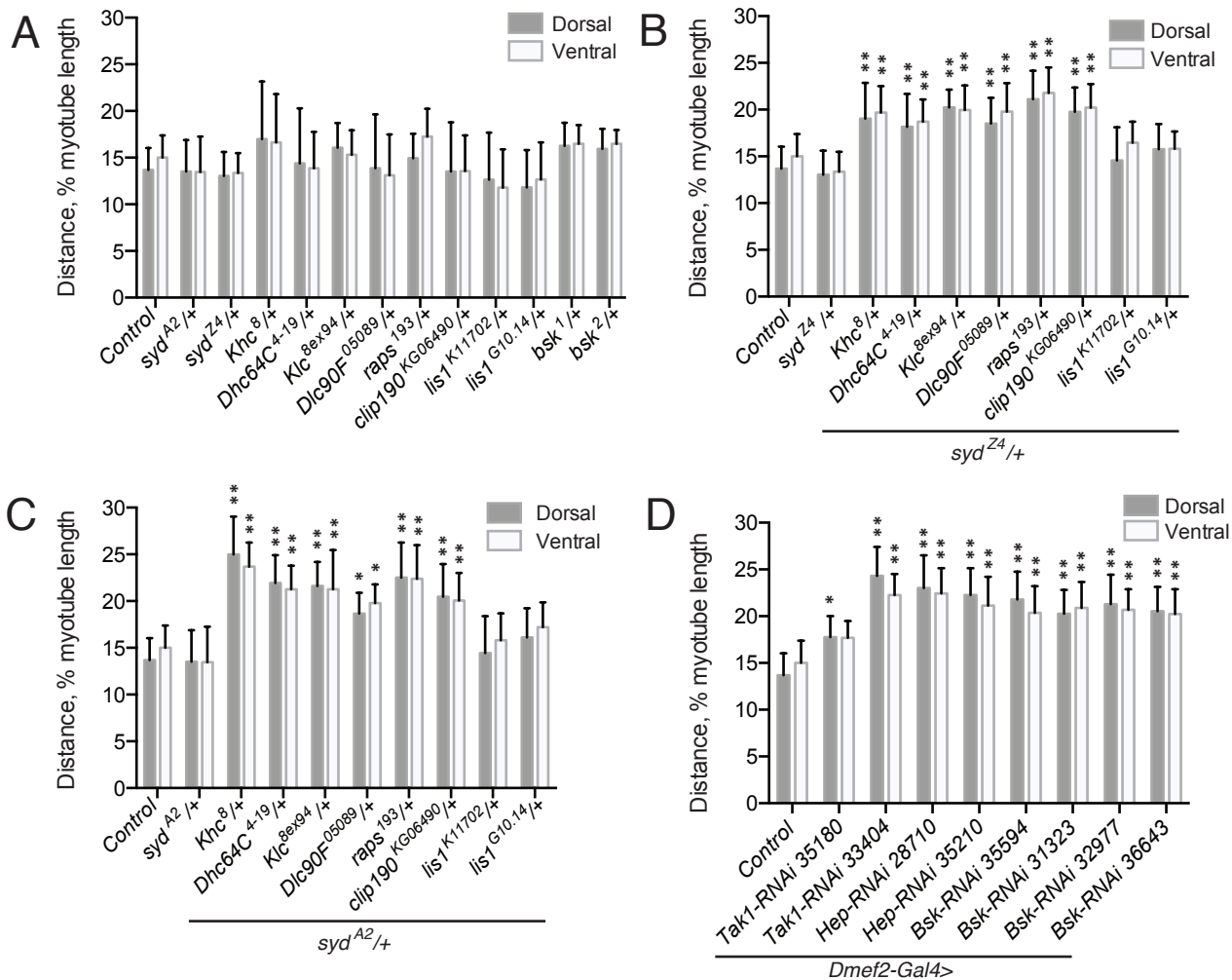

Schulman et al., Supplemental Figure 3

Supplement: S3 Figure — Supporting genetic assays. A-D) Analysis of myonuclear position by measuring the shortest distance between the LT muscle ends (Dorsal, grey; Ventral, white) and the nearest nucleus normalized for muscle length using confocal projection imagess of stage 16 embryos immunostained for Tropomyosin/muscles (green) and dsRed/nuclei (red). A) Single heterozygous embryos for the listed alleles. B) Doubly heterozygous embryos of sydZ4 and factors involved in muscle development in which the sydZ4 allele was paternally provided. C) Identical to B using the sydA2 allele. D) Multiple UAS-RNAi constructs targeting JNK signaling module components expressed in muscles using Dmef2-Gal4. For each genotype in A–D, all four LT muscles were measured in each of three hemisegments from ten embryos from at least three independent experiments. All error bars represent standard deviation. *, p<0.05; **, p<0.01 compared to controls (Student's t-test and ANOVA assessment). (PDF) [file pgen.1004880.s003.pdf]

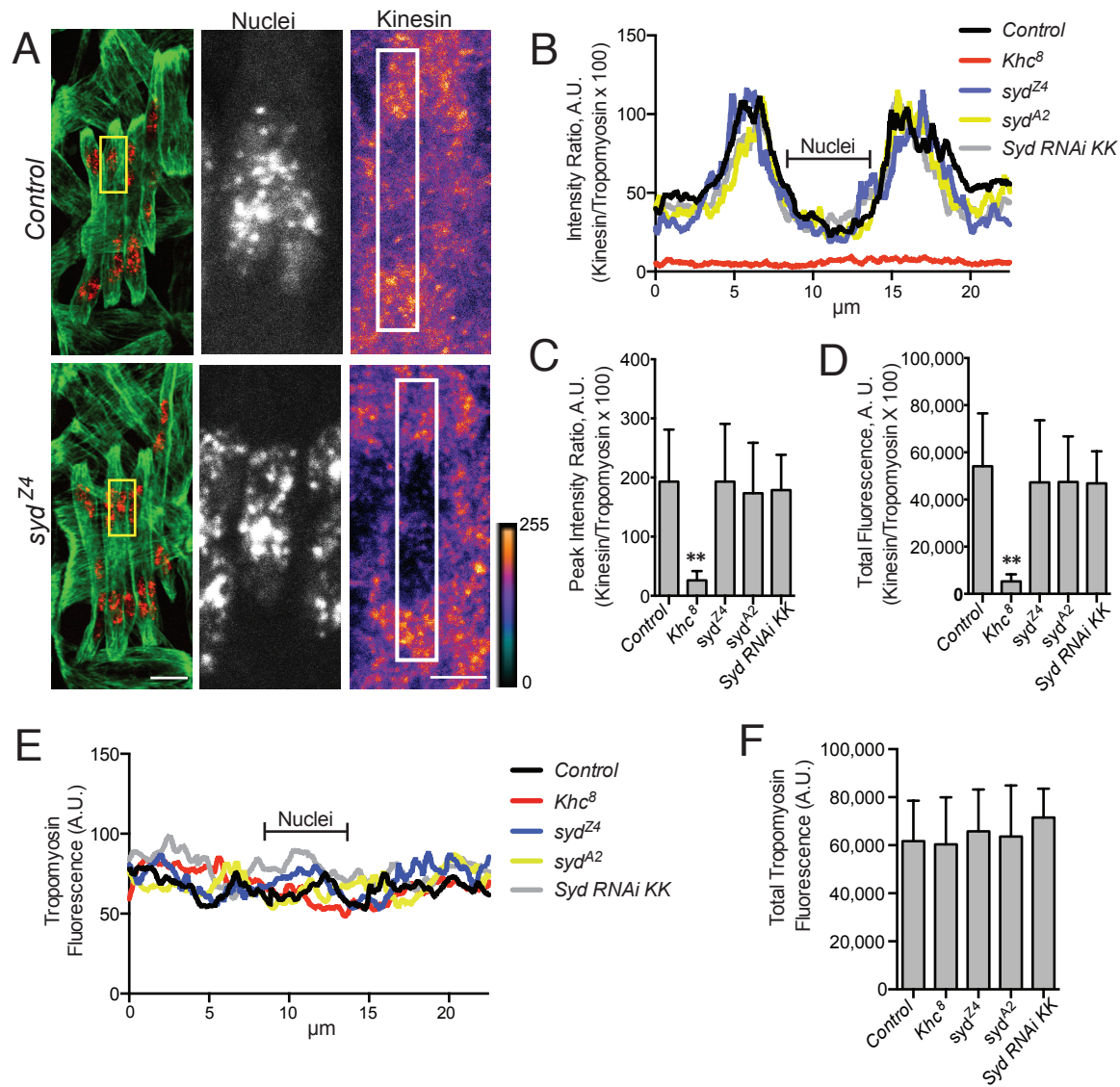

Supplement: S4 Figure — Syd is not required for Kinesin localization. A) (Left) Immunofluorescence projection images of the LT muscles in one hemisegment of stage 16 embryos. Green, Tropomyosin/muscles; red, dsRed/nuclei. Yellow boxes identify regions of higher magnification shown to the right. Scale bar, 10 µm. (Middle, Right) High magnification views of Kinesin immunofluorescence near the nuclei (grayscale). Kinesin shown as a heatmap to highlight regions of accumulation (right). Scale of relative intensities shown at lower right. White boxes denote regions used for immunofluorescence analysis of Kinesin localization. Scale bar, 5 µm. B) Intensity profile of Kinesin immunofluorescence relative to Tropomyosin immunofluorescence plotted as a function of position. Zero/left, corresponds to the top end of white boxed regions in A; Right, corresponds to the lower end of white boxed regions in A. C) Average peak intensity of Kinesin immunofluorescence. D) Average total fluorescence of Kinesin determined by calculating the area under the curves in B. E–F) Validation for the use of Tropomyosin, which is similar in all genotypes, as a reference/internal immunostaining control for comparing changes in Kinesin immunofluorescence in B–D. E) Immunofluorescence intensity profile for Tropomyosin plotted as a function of position across the same cellular region examined in B. F) Average total fluorescence of Tropomyosin determined by calculating the area under the curves in E. For each genotype A–F, two LT muscles were measured in each of three hemisegments from ten embryos from at least three independent experiments. All error bars represent standard deviation. **, p<0.01 compared to controls (Student's t-test and ANOVA assessment). A.U., arbitrary units. (PDF) [file pgen.1004880.s004.pdf]

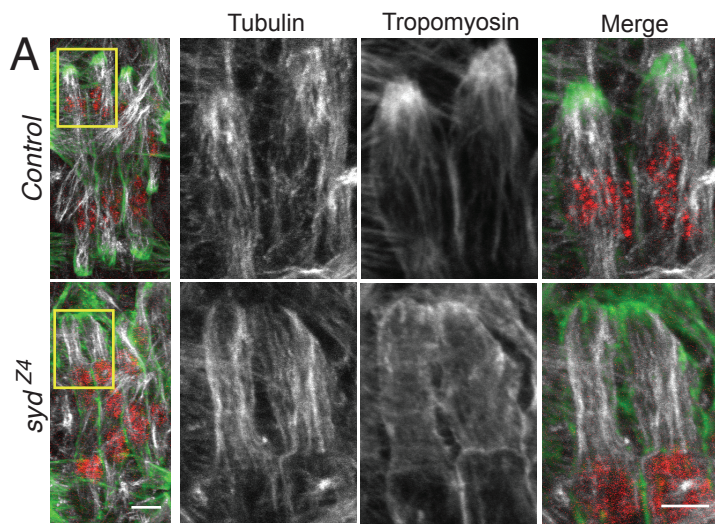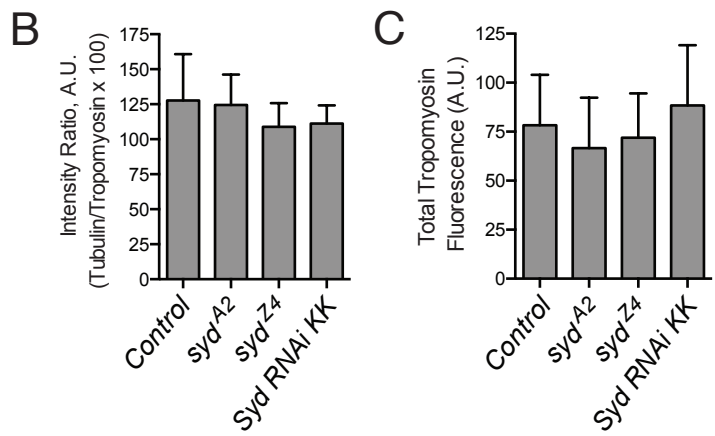

Supplement: S5 Figure — Syd does not affect gross microtubule organization. A) (Left) Immunofluorescence projection images of the LT muscles in one hemisegment of stage 16 embryos. Green, Tropomyosin/muscles; red, dsRed/nuclei; white, Tubulin/microtubules. Yellow boxes identify regions of higher magnification shown to the right. Scale bar, 10 µm. (Right three panels) High magnification views of Tropomyosin and Tubulin immunofluorescence shown in grayscale and merged (colors as in A). Scale bar, 5 µm. B) Average Tubulin intensity relative to Tropomyosin intensity in the distal 2 µm of the muscle fiber, demonstrating that microtubules reach the muscle end in all genotypes. C) Validation for the use of Tropomyosin, which is similar in all genotypes, as a reference/internal immunostaining control for comparing changes in Tubulin immunofluorescence in B. Values represent total Tropomyosin immunofluorescence detected in the distal 2 µm of the muscle fiber. For each genotype in A–C, two LT muscles were measured in each of three hemisegments from ten embryos from at least three independent experiments. All error bars represent standard deviation. Values are not significant by Student's t-test or ANOVA assessment. A.U., arbitrary units. (PDF) [file pgen.1004880.s005.pdf]

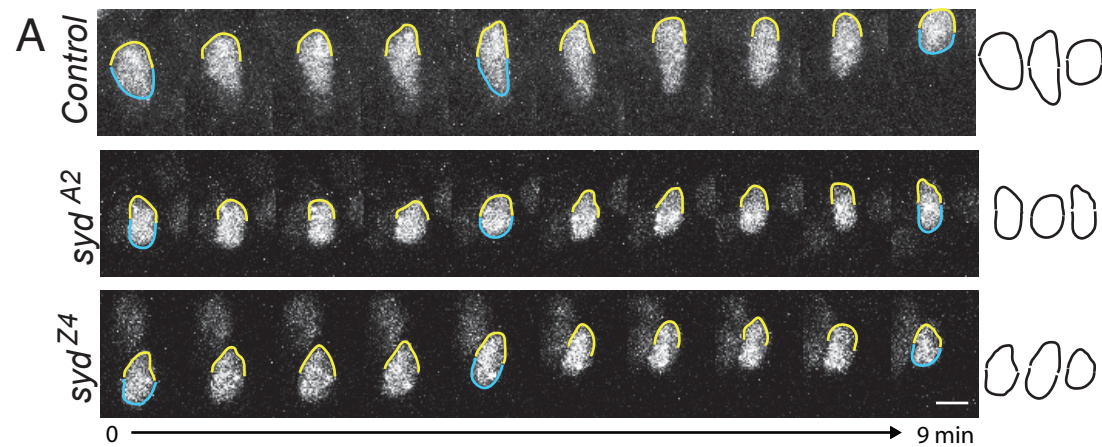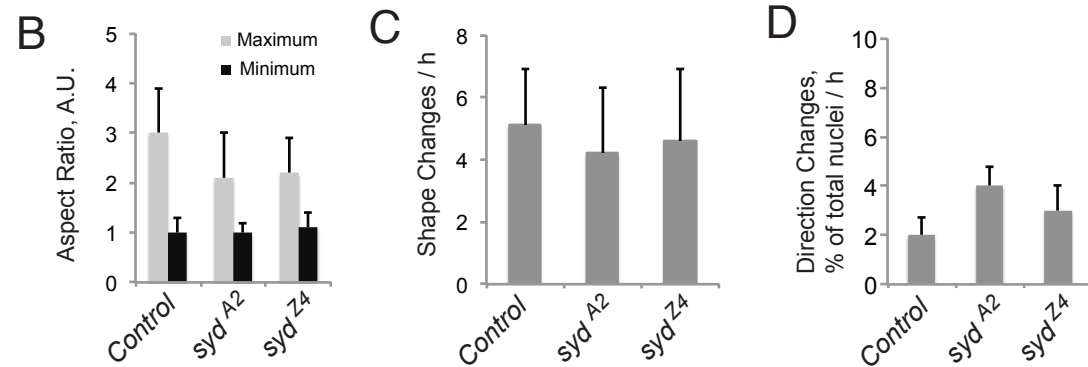

Supplement: S6 Figure — Syd does not impact Kinesin- and Dynein-dependent nuclear shape changes and translocation dynamics. A) Kymographs of individual translocating myonuclei in the indicated genotypes with all nuclei moving in the upward direction. Yellow lines indicate the leading edge of the myonucleus and highlight leading edge dynamics during translocation. Blue lines complete the perimeter of the nuclei at individual time-points and are combined with the yellow lines to produce the cartooned shapes to the right to highlight nuclear shape changes over time. Scale bar, 2 µm. B) Histogram indicating the average maximum and minimum aspect ratio of myonuclei as they translocated for between 20 min and 1 h. Maximums and minimums were compared individually. C) Histogram indicating the number of shape changes individual myonuclei experience per hour. Values were calculated by following individual myonuclei as they moved for between 20 min and 1 hour. D) Histogram indicating the percentage of total nuclei that change direction per hour in indicated genotypes. For each genotype in A-D, 100 individual LT muscle nuclei from at least three hemisegments from five different embryos from at least three independent experiments were measured. All error bars represent standard deviation. Values are not significant by Student's t-test or ANOVA assessment. (PDF) [file pgen.1004880.s006.pdf]

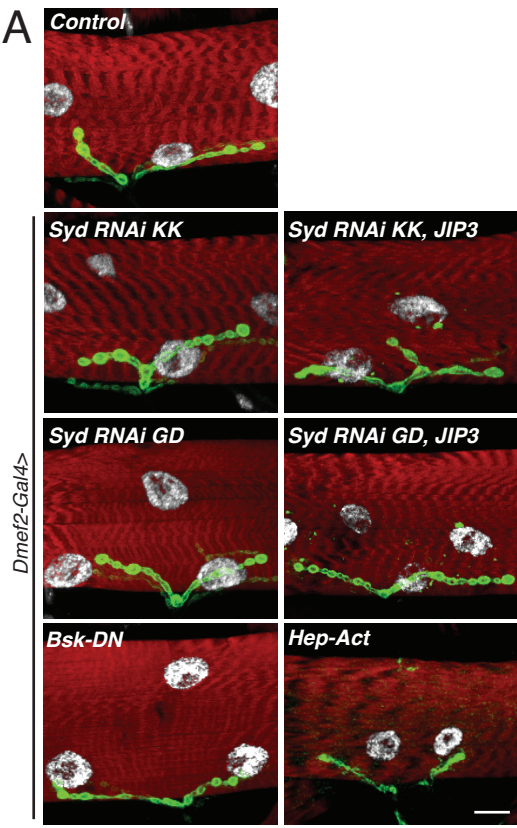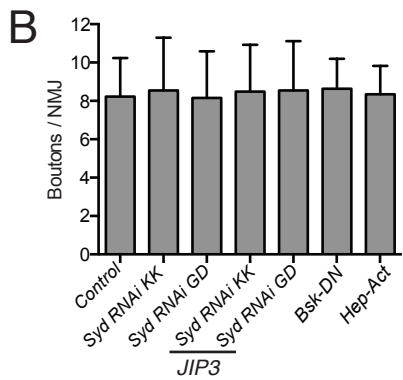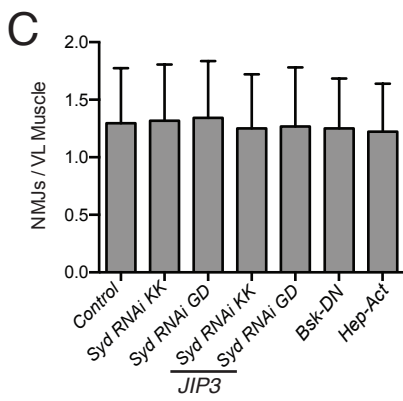

Schulman et al., Supplemental Figure 7

Supplement: S7 Figure — NMJs are unaffected in syd mutants. A) Immunofluorescence projection images of the VL muscles in dissected L3 Drosophila larvae. Red, Phalloidin/sarcomeres; green, Discs large/NMJs; white, Hoescht/nuclei. Scale bar, 20 µm. B) Number of boutons per NMJ as determined by Discs large staining. C) Number of NMJs/innervations per muscle. For each genotype in A–C, three VL muscles (1, 2, and 4) in six hemisegments from five dissected larvae from at least three independent experiments were measured/counted. All error bars represent standard deviation. Values are not significant by Student's t-test or ANOVA assessment. (PDF) [file pgen.1004880.s007.pdf]
