# Supplementary material for: Syd/JIP3 and JNK Signaling Are Required for Myonuclear Positioning and Muscle Function
Source: PLoS Genet. 2014 Dec 18;10(12):e1004880. doi: 10.1371/journal.pgen.1004880 (PMC4270490; doi:10.1371/journal.pgen.1004880)
Supplement: S2 Figure — Syd protein and DNA alignments. A) Protein alignment of Syd to mammalian JIP3 using Clustal Omega. Amino acid number indicated at right. Red box indicates the conserved region recognized by the C-terminal Syd/JIP3 antibody used in main text Fig. 1 to detect both proteins. *, denotes identical residues; dots, similar residues. B–E) Syd or mammalian JIP3 DNA sequences aligned to either Syd-RNAi KK or Syd-RNAi GD using Clustal W2. Nucleotide number indicated at right. *, denotes nucleotide matches. B) Alignment of Syd to Syd-RNAi KK. C) Alignment of Syd to Syd-RNAi GD. D) Alignment of JIP3 to Syd-RNAi KK. E) Alignment of JIP3 to Syd-RNAi GD. (PDF) [file pgen.1004880.s002.pdf]

A

```
JIP3     TMQIEKSFDAHPRRESQVRQLAWIGDGVWVSIRLDSTLRLYHAHTHQHLQDVDIEPYVSK 1133
Syd      SLNIVHSLDAHPRKESQVRQMAATGAGVWVSIRLDSTLRLYNTHTFEHKQDVDIEPYVSK 1080
         :::* :*:*****:******:*  * ***************.:**.:* ***********
```

B

```
Syd              ACCAAATCGCTGAACCCCATACTGGAGACCAAGGATCGTGATGAACCGGCAATGAGCTCT 2640
Syd-RNAi-KK      ----AATCGCTGAACCCCATACTGGAGACCAAGGATCGTGATGAACCGGCAATGAGCTCT 56
                     ********************************************************

Syd              GTAGGTCCAACAATGTGGCTGGGCGCCCAGGATGGCTGGCTGTATGTGCACAGCAGCGTG 2700
Syd-RNAi-KK      GTAGGTCCAACAATGTGGCTGGGCGCCCAGGATGGCTGGCTGTATGTGCACAGCAGCGTG 116
                 ************************************************************

Syd              GGAAGGTGGCACGAGTGCCTGCACCGAGTTCTCCTGCCGGATGCTGTGCTAGCGATTGTA 2760
Syd-RNAi-KK      GGAAGGTGGCACGAGTGCCTGCACCGAGTTCTCCTGCCGGATGCTGTGCTAGCGATTGTA 176
                 ************************************************************

Syd              CACGTGGAGGCGAGGGTCGTTGTGGCGCTGGCCAATGCCCAACTGGCTGTGTTCCGCCGC 2820
Syd-RNAi-KK      CACGTGGAGGCGAGGGTCGTTGTGGCGCTGGCCAATGCCCAACTGGCTGTGTTCCGCCGC 236
                 ************************************************************

Syd              CAGACAGACGGCCAATGGGATCTGAATAGCTATCACCTGGTGACGCTCGGTGATCGCAAC 2880
Syd-RNAi-KK      CAGACAGACGGCCAATGGGATCTGAATAGCTATCACCTGGTGACGCTCGGTGATCGCAAC 296
                 ************************************************************

Syd              CATTCGATACGTTGCCTCTGTGTGGCTGGCGAGCGCATTTGGGCCGCTCACCGCAACAAG 2940
Syd-RNAi-KK      CATTCGATACGTTGCCTC------------------------------------------ 314
                 ******************
```

C

```
Syd              ATAAGTGCAAGGAGGCGGGAACAGTACCGCCAGCTACGAGCCCATGTCCAAAAGGAGGAT 1740
Syd-RNAi-GD      ------------------------------CAGCTACGAGCCCATGTCCAAAAGGAGGAT 30
                                               ******************************

Syd              GGGCGGCTGCATGCCTACGGTTGGAGTCTGCCGATTAACAAGGCCAGCCAGGAGGCGAAT 1800
Syd-RNAi-GD      GGGCGGCTGCATGCCTACGGTTGGAGTCTGCCGATTAACAAGGCCAGCCAGGAGGCGAAT 90
                 ************************************************************

Syd              CCCAATCGGCATTCAGGAGGTGTACCGGTACCCGTATATTGTAATCCCCTGGCGGAGGCA 1860
Syd-RNAi-GD      CCCAATCGGCATTCAGGAGGTGTACCGGTACCCGTATATTGTAATCCCCTGGCGGAGGCA 150
                 ************************************************************

Syd              TCGCCCCACATGAAGGTATTTTGTGCGGCAGGCGTTAACCTACACGGCGGGTTCACAAAG 1920
Syd-RNAi-GD      TCGCCCCACATGAAGGTATTTTGTGCGGCAGGCGTTAACCTACACGGCGGGTTCACAAAG 210
                 ************************************************************

Syd              AATGGGCAATCACTGATACCTGCCAACTCACCATATGCTCCGAAATCTACGCTCAAGATA 1980
Syd-RNAi-GD      AATGGGCAATCACTGATACCTGCCAACTCACCATATGCTCCGAAATCTACGCTCAAGATA 270
                 ************************************************************

Syd              GCTGAGATAACCAGTCCCACGGCGGATCAGAGCATGGAGGCTTTGGACAGGCAGATGGCG 2040
Syd-RNAi-GD      GCTGAGATAACCAGTCCCACGGCGGATCAGAGCATGGAGGCTTTGGACAGGCAGATGGCG 330
                 ************************************************************

Syd              AGAGTCAGTCTGGAGACACTGGAGCCCGAGACGCAACTCAGTTCGTTCGTATGGATATGC 2100
Syd-RNAi-GD      A----------------------------------------------------------- 331
                 *
```

D

```
JIP3             GATTCTACCCTTCGGCTCTACCATGCTCACACCCACCAGCACCTGCAGGATGTGGACATT 3420
Syd-RNAi-KK      ---------------------------------------CTCCTGCCGGATG-------- 159
                                                        * ***** *****

JIP3             GAGCCCTATGTTAGCAAGATGCTAGGAACCGGCAAGCTGGGCTTCTCCTTCGTGCGCATC 3480
Syd-RNAi-KK      -----CTGTGCTAGCGA--------------------------------TTGTACACGT- 181
                      ** ** **** *                                * ** * * *

JIP3             ACAGCCTTACTCATTGCAGGCAACCGTCTGTGGGTGGGCACTGGCAATGGGGTTGTCATC 3540
Syd-RNAi-KK      ---------------GGAGGCGA--------------------------GGGTCGT---- 196
                                * **** *                          **** **

JIP3             TCCATCCCCTTGACTGAGACTGTGGTCCTGCATCGAGGCCAGCTCCTAGGGCTCCGAGCC 3600
Syd-RNAi-KK      --------------------TGTGGCGCTG---------------------------GCC 209
                                     *****  ***                           ***

JIP3             AACAAGACATCCCCAACATCTGGGGAGGGGACCCGCCCAGGGGGCATCATCCATGTGTAT 3660
Syd-RNAi-KK      AAT-------------------------------GCCCA--------------------- 217
                 **                                *****

JIP3             GGGGACGACAGCAGTGACAAGGCCGCCAGTAGTTTCATCCCCTACTGCTCCATGGCACAG 3720
Syd-RNAi-KK      -------------------------------------------ACTG------------- 221
                                                            ****

JIP3             GCTCAGCTTTGCTTCCATGGGCACCGTGATGCTGTCAAATTCTTTGTCTCTGTGCCAGGA 3780
Syd-RNAi-KK      -----GCTGTGTTCC---------------GCCGCCAGAC-------------------- 241
                      *** ** * *               ** * ** *

JIP3             AATGTGCTGGCCACTCTCAATGGCAGTGTGCTAGACAGCCCATCAGAGGGCCCTGGGCCT 3840
Syd-RNAi-KK      --------------------------------AGACGGCCAAT----------------- 252
                                                 **** *** **

JIP3             GCTGCACCCGTTGCAGATGCTGAGGGCCAGAAGTTGAAGAATGCACTGGTGCTGAGTGGT 3900
Syd-RNAi-KK      -------------------------------------GGGAT---------CTGAATAGC 266
                                                       * **          **** * *

JIP3             GGTGAAGGTTACATTGACTTCCGTATCGGAGACGGAGAGGATGATGAAACTGAGGAATGT 3960
Syd-RNAi-KK      -----------------------TATC----AC--------------------------- 272
                                        ****    **

JIP3             GCCGGGGACGTGAACCAGACAAAGCCCTCGTTGTCCAAGGATGAGCGCACGCACATCATC 4020
Syd-RNAi-KK      -CTGGTGACG----------------CTCG----------GTGATCGCA-----ACCATT 300
                  * ** ****                ****          *** ****     * ***

JIP3             GTGTGGCAGGTGTCCTACACCCCTGAGTGAGACCCTGTCCTACCTGATGCCAACTGTACA 4080
Syd-RNAi-KK      -----------------------------------------------------CGATACG 307
                                                                      *  ***
```

E

```
JIP3             CTAGACAGTGATGTGAACCCTGAAGATTCAGGTGCTGATGGTGTGCTGGCTGGCATCACC 2580
Syd-RNAi-GD      CCCCACA-------------TGAAGGT----------ATTTGTGC-GGCAGGCGTTAAC 189
                 *   ***             ***** *          **  ***** *** *** * * *

JIP3             CTGGTGGGGTGTGCTACCCGCTGCAATGTTCCACGTAGCAACTGTTCCTCACGAGGAGAC 2640
Syd-RNAi-GD      CT--------------------------------------------------------AC 193
                 **                                                        **

JIP3             ACCCCAGTACTGGACAAGGGGCAGGGGGATGTGGCGACCACTGCCAATGGGAAGGTCAAC 2700
Syd-RNAi-GD      AC-----------------GGCGGG----------------------------------- 201
                 **                 *** **

JIP3             CCGTCCCAATCCACAGAAGAAGCCACAGAAGCCACGGAGGTGCCAGACCCTGGTCCCAGC 2760
Syd-RNAi-GD      ---------TTCACA-AAGAA-----------------------------TGG------- 215
                          * **** *****                             ***

JIP3             GAGTCAGAAGCAACGACAGTCCGGCCCGGGCCTCTCACAGAGCATGTCTTTACTGACCCA 2820
Syd-RNAi-GD      ---------GCAA------------------------------------TCACTGAT--- 227
                          ****                                    * *****

JIP3             GCACCCACCCCATCCTCCAGCACCCAGCCTGCCAGTGAGAATGGGTCAGAGTCCAATGGC 2880
Syd-RNAi-GD      --------------------------ACCTGCCA-------------------------- 235
                                            *******

JIP3             GCACCCACTATGTGGCTAGGAGCCCAGAATGGCTGGCTCTATGTGCATTCAGCGGTAGCC 3000
Syd-RNAi-GD      --ACTCACCATAT--------GCTCCGAA------------------------------- 254
                   ** *** ** *        ** * ***

JIP3             AACTGGAAGAAGTGTCTGCACTCCATCAAGCTAAAAGACTCTGTGCTGAGCCTGGTGCAT 3060
Syd-RNAi-GD      -------------ATCTACGCTC----AAGATA-----------GCTGAG---------- 276
                               *** * ***    *** **           ******

JIP3             GTCAAAGGCCGAGTGCTGGTAGCTCTTGCAGATGGGACCCTGGCTATCTTCCATCGTGGA 3120
Syd-RNAi-GD      ------------------ATAAC-----CAG-----------------TCCCAC------ 290
                                    ** *     ***                 * ***

JIP3             GAGGATGGCCAGTGGGACCTGAGCAACTACCACCTAATGGACCTGGGCCACCCACACCAC 3180
Syd-RNAi-GD      ------GGC-----GGATCAGAGCA------------TGGA----GGC------------ 311
                       ***     *** * *****            ****    ***

JIP3             TCCATCCGTTGCATGGCTGTTGTGAATGACCGAGTTTGGTGTGGCTACAAGAACAAGGTG 3240
Syd-RNAi-GD      ----------------------------------TTTGG-------------ACAGG--- 321
                                                   *****             *** *

JIP3             CATGTTATCCAGCCCAAGACAATGCAGATTGAGAAATCATTTGATGCCCACCCAAGGCGG 3300
Syd-RNAi-GD      ------------------------CAGAT--------------------------GGCGA 331
                                         *****                          ****
```

Schulman et al., Supplemental Figure 2
